# Disease modeling by efficient genome editing using a near PAM-less base editor in vivo

**DOI:** 10.1038/s41467-022-31172-z

**Published:** 2022-06-15

**Authors:** Marion Rosello, Malo Serafini, Luca Mignani, Dario Finazzi, Carine Giovannangeli, Marina C. Mione, Jean-Paul Concordet, Filippo Del Bene

**Affiliations:** 1 Sorbonne Université, INSERM U968, CNRS UMR 7210, Institut de la Vision, Paris, France; 2 Department of Molecular and Translational Medicine, University of Brescia, Brescia, Italy; 3 Muséum National d’Histoire Naturelle, INSERM U1154, CNRS UMR 7196, Paris, France; 4 Department of Cellular, Computational and Integrative Biology – CIBIO, University of Trento, Trento, Italy

**Keywords:** Genetic engineering, Genetic engineering

## Abstract

Base Editors are emerging as an innovative technology to introduce point mutations in complex genomes. So far, the requirement of an NGG Protospacer Adjacent Motif (PAM) at a suitable position often limits the base editing possibility to model human pathological mutations in animals. Here we show that, using the CBE4max-SpRY variant recognizing nearly all PAM sequences, we could introduce point mutations for the first time in an animal model with high efficiency, thus drastically increasing the base editing possibilities. With this near PAM-less base editor we could simultaneously mutate several genes and we developed a co-selection method to identify the most edited embryos based on a simple visual screening. Finally, we apply our method to create a zebrafish model for melanoma predisposition based on the simultaneous base editing of multiple genes. Altogether, our results considerably expand the Base Editor application to introduce human disease-causing mutations in zebrafish.

## Abstract (web summary)

Base Editors are emerging as an innovative technology to introduce point mutations in complex genomes. Here the authors describe a near PAM-less base editor and its application in zebrafish to efficiently create disease models harbouring specific point mutations.

## Introduction

The CRISPR (Clustered Regularly Interspaced Short Palindromic Repeats)/Cas9 system is a very powerful gene-editing tool to perform mutagenesis in zebrafish^1^. The sgRNA-Cas9 complex first binds to target DNA through a Protospacer Adjacent Motif (PAM) and Cas9 next cleaves DNA upon stable annealing of the sgRNA spacer sequence to the sequence immediately upstream of the PAM. The NGG PAM sequence is thus required for the *Sp*Cas9 protein to introduce a DNA double-strand break (DSB). This technique is now extensively used in zebrafish to produce knock-out alleles^2^. Additionally, recent studies showed that exogenous DNA and single nucleotide polymorphisms (SNPs) can be introduced in the genome using CRISPR/Cas9-mediated homology-directed repair (HDR) with variable efficiency^3–5^. In order to complement these HDR-based strategies to introduce specific point mutation, second-generation gene-editing tools called base editors (BEs) have recently been developed. The Cytidine Base Editor (CBE) is composed of a Cas9(D10A) nickase fused to a cytidine deaminase and converts C-to-T bases in a restricted window of 13–19 nucleotides (nt) upstream of the PAM sequence without inducing double-stranded DNA cleavage^6–8^. In zebrafish, several CBE variants have been shown to work with different gene editing efficiencies^9–12^. In a previous study, we tested several CBE variants and we were able to reach a C-to-T conversion efficacy of 91% in zebrafish, showing many applications from signaling pathway activation to human disease modeling^12^. It has been shown that potentially all C bases within the PAM [−19, −13] bp window can be edited with these tools, although a higher efficiency was generally achieved for the Cs located in the middle of this editing window, highlighting the importance of the C distance to the PAM for efficient base editing^13^. Therefore, the restriction of the base modification to the PAM-dependent window is still a critical intrinsic limitation to the base editor techniques. Due to this restriction, these tools cannot be applied to any gene and any mutation of interest. To overcome this limitation, extensive works have been done in cultured cells to engineer CBEs recognizing other PAM sequences than the classical NGG and thereby significantly expand the range of C bases that can be converted. Based on the technological advances made in cultured cells, we tested several recently developed CBEs in zebrafish to overcome the PAM limitation. Among them, the most recent and flexible variant is the CBE4max-SpRY, reported as a near PAM-less base editor recognizing almost all PAM sequences in cultured cells^14^. Here we established the CBE4max-SpRY CBE variant for the first time in an animal model. Using this variant, we were able to perform in zebrafish C-to-T conversions at an unprecedented high efficiency. We show that the CBE4max-SpRY converts C bases efficiently using NRN PAMs in zebrafish and that it is possible to mutate several genes using NGN and NAN PAMs at the same time, increasing drastically the base editing possibilities. We also show base editing using NYN PAMs, but with a lower efficiency. Based on these results, we developed a co-selection method to phenotypically identify the most edited embryos following the CBE4max-SpRY, by co-targeting the *tyrosinase* or *slc45a2* genes and selecting larvae based on a lack of pigmentation. Finally, using this approach, we could simultaneously introduce a loss-of-function mutation in a tumor suppressor gene together with a gain-of-function mutation in an endogenous oncogene. We targeted *tp53* tumor suppressor and *nras* oncogene and generated a zebrafish model with an abnormal melanocyte growth that is an hallmark of melanoma formation susceptibility^15^, without over-expressing mutated oncogenes which has been the main strategy used in zebrafish to model cancer so far^16–19^.

## Results

### Evaluation of several CBE variants recognizing NGN PAM in zebrafish

Base editing requires the presence of a PAM at 13–19 bp downstream of the targeted C base. This limitation is critical and often makes CBEs unable to introduce the desired point mutation in the genome. To overcome this limitation particularly important when trying to model disease-causing mutations in animal models, we first tested three different CBE variants recognizing the NGN PAM: xCas9-BE4^20^, CBE4max-SpG^14^ and CBE4max-SpRY^14^. First, we injected into one-cell stage embryos the *xCas9-BE4* mRNA with the tp53 Q170* sgRNA, a sgRNA with which we previously got up to 86% efficiency using BE4-gam^12^. No base conversion was detected by Sanger Sequencing of PCR products of the target region. We next analyzed the efficacies of the recent CBE4max-SpG and CBE4max-SpRY variants in zebrafish by injecting into one-cell stage embryos sgRNAs acting upstream of an NGG PAM that we previously found to be efficient with the original CBEs for 5 different loci^12^ (Table 1). We sequenced the target regions from 8 single embryos and a pool of 30 embryos for each gene targeted independently by the AncBE4max, the CBE4max-SpG and the CBE4max-SpRY. We detected C-to-T conversion using the CBE4max-SpG only when targeting *cbl* gene for 1 out of 8 single injected embryos whereas we got C-to-T conversions for the 5 loci and for the majority of the injected embryos analyzed with up to 87% of efficiency using CBE4max-SpRY with Sanger sequencing analyses (Table 1). Although working efficiently, the CBE4max-SpRY seems overall to remain less efficient than the classical AncBE4max. The CBE4max-SpRY has been reported as a near PAM-less base editor in cultured cells, with a higher efficacy using NRN PAMs^14^. We thus next chose to analyze its flexibility by targeting genes implicated in the formation of pigments in order to have an easy phenotypic read out of the base conversion efficiency.

**Table 1:** Efficient base editing using CBE4max-SpRY.

	CBE used	Number of edited embryos	Emb.1			Emb.2			Emb.3			Emb.4			Emb.5			Emb.6			Emb.7			Emb.8			Pool of 30 emb.		
bap1 (Q173*)	AncBE4max	6/8	41%			40%			34%			32%			11%			9%			n.d.			n.d.			-		
	CBE4max-SpG	0/8	n.d.			n.d.			n.d.			n.d.			n.d.			n.d.			n.d.			n.d.			n.d.		
	CBE4max-SpRY	6/8	87%			72%			45%			32%			26%			9%			n.d.			n.d.			34%		
tek (Q64*)	AncBE4max	8/8	C1417%	C13 n.d.		C14 56%	C13 32%		C14 28%	C13 5%		C14 51%	C13 9%		C14 21%	C13 n.d.		C14 26%	C13 19%		C14 51%	C13 22%		C14 29%	C13 n.d.		C14 14%	C13 19%	
	CBE4max-SpG	0/8	C14 n.d.	C13 n.d.		C14 n.d.	C13 n.d.		C14 n.d.	C13 n.d.		C14 n.d.	C13 n.d.		C14 n.d.	C13 n.d.		C14 n.d.	C13 n.d.		C14 n.d.	C13 n.d.		C14 n.d.	C13 n.d.		C14 n.d.	C13 n.d.	
	CBE4max-SpRY	5/8	C14 20%	C13 7%		C14 12%	C13 4%		C14 11%	C13 4%		C14 10%	C13 3%		C14 8%	C13 3%		C14 n.d.	C13 n.d.		C14 n.d.	C13 n.d.		C14 n.d.	C13 n.d.		C14 9%	C13 3%	
cbl (W577*)	AncBE4max	8/8	C16 100%	C15 56%		C16 82%	C15 63%		C16 80%	C15 46%		C16 70%	C15 49%		C16 60%	C15 33%		C16 54%	C15 39%		C16 42%	C15 4%		C16 34%	C15 22%		C16 34%	C15 26%	
	CBE4max-SpG	1/8	C16 9%	C15 8%		C16 n.d.	C15 n.d.		C16 n.d.	C15 n.d.		C16 n.d.	C15 n.d.		C16 n.d.	C15 n.d.		C16 n.d.	C15 n.d.		C16 n.d.	C15 n.d.		C16 n.d.	C15 n.d.		C16 n.d.	C15 n.d.	
	CBE4max-SpRY	8/8	C16 17%	C15 21%		C16 18%	C15 20%		C16 17%	C15 13%		C16 11%	C15 14%		C16 9%	C15 14%		C16 9%	C15 13%		C16 8%	C15 9%		C16 6%	C15 6%		C16 10%	C15 15%	
dmd (Q8*)	AncBE4max	8/8	15%			27%			12%			13%			19%			9%			12%			8%			15%		
	CBE4max-SpG	0/8	n.d.			n.d.			n.d.			n.d.			n.d.			n.d.			n.d.			n.d.			n.d.		
	CBE4max-SpRY	7/8	17%			14%			14%			10%			10%			9%			8%			n.d.			13%		
rb1 (W63*)	AncBE4max	5/8	C19 n.d.	C17 n.d.	C16 55%	C19 n.d.	C17 72%	C16 32%	C19 n.d.	C17 44%	C16 7%	C19 n.d.	C17 76%	C16 58%	C19 n.d.	C17 5%	C16 35%	C19 n.d.	C17 n.d.	C16 n.d.	C19 n.d.	C17 n.d.	C16 n.d.	C19 n.d.	C17 n.d.	C16 n.d.	C19 n.d.	C17 37%	C16 16%
	CBE4max-SpG	0/8	C19 n.d.	C17 n.d.	C16 n.d.	C19 n.d.	C17 n.d.	C16 n.d.	C19 n.d.	C17 n.d.	C16 n.d.	C19 n.d.	C17 n.d.	C16 n.d.	C19 n.d.	C17 n.d.	C16 n.d.	C19 n.d.	C17 n.d.	C16 n.d.	C19 n.d.	C17 n.d.	C16 n.d.	C19 n.d.	C17 n.d.	C16 n.d.	C19 n.d.	C17 n.d.	C16 n.d.
	CBE4max-SpRY	4/8	C19 n.d.	C17 33%	C16 24%	C19 n.d.	C17 22%	C16 n.d.	C19 n.d.	C17 24%	C16 19%	C19 n.d.	C17 31%	C16 24%	C19 n.d.	C17 n.d.	C16 n.d.	C19 n.d.	C17 n.d.	C16 n.d.	C19 n.d.	C17 n.d.	C16 n.d.	C19 n.d.	C17 n.d.	C16 n.d.	C19 n.d.	C17 n.d.	C16 n.d.

Base editing efficiency quantifications on 8 single and a pool of 30 embryos of 1 dpf randomly chosen after the injection of indicated *CBE* mRNA and sgRNA using NGG PAMs. The base editing efficiency varies between 0 and 100% in a single embryo depending on the targeted locus, the sgRNA, and the CBE used. Each number indicated next to each C base refers to the distance of the C from the PAM (in base pair). Base editing efficiencies were quantified using EditR tool^42^. N.d. = no base editing detected using EditR tool.

### Albino phenotype generation in F0 embryos using the CBE4max-SpRY and a NAN PAM

**Fig. 1:**
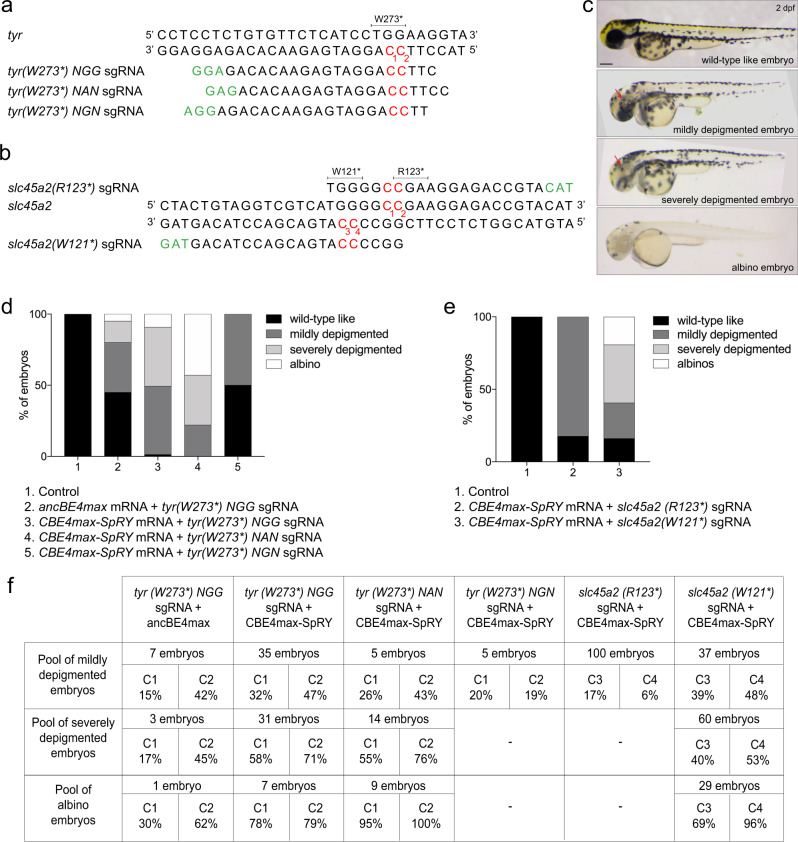
Efficient base conversion generates albino F0 embryos with CBE4max-SpRY and a NAN PAM. **a** Targeted genomic sequence of the *tyrosinase* gene and the 3 different sgRNAs used to introduce the W273* mutation. **b** Targeted genomic sequence of the *slc45a2* gene and the 2 different sgRNAs used to introduce the W121* or R123* mutations. a, b. For each sgRNA, the targeted Cs are in red and the PAM sequence is in green. **c** Lateral view of representative 2 days post-fertilization (dpf) embryos showing different severity of pigmentation defects (wild-type like, mildly depigmented, severely depigmented, and albino embryos). Scale bar = 100 µm. **d** Proportion of the 4 groups based on the pigmentation defects described in Fig. 1c for each injection: the *AncBE4max* mRNA and the tyr(W273*)NGG sgRNA (column 2, 19 embryos in total), the *CBE4max-SpRY* mRNA and the tyr(W273*)NGG sgRNA (column 3, 74 embryos in total), the *CBE4max-SpRY* mRNA and the tyr(W273*)NAN sgRNA (column 4, 28 embryos in total), the *CBE4max-SpRY* mRNA and the tyr(W273*)NGN sgRNA (column 5, 10 embryos in total). **e** Proportion of the 4 groups based on the pigmentation defects described in **c** for each injection: the *CBE4max-SpRY* mRNA with the slc45a2(R123*) sgRNA (column 2, 100 embryos in total) or with the slc45a2(R121*) sgRNA (column 3, 126 embryos in total). **f** C-to-T conversion efficiency for each targeted Cs and each pool of embryos showing pigmentation defects presented in **d**, **e**. The efficiencies have been calculated using EditR tool^42^ and chromatograms from Sanger sequencing made on entire PCR products.

**Fig. 2:**
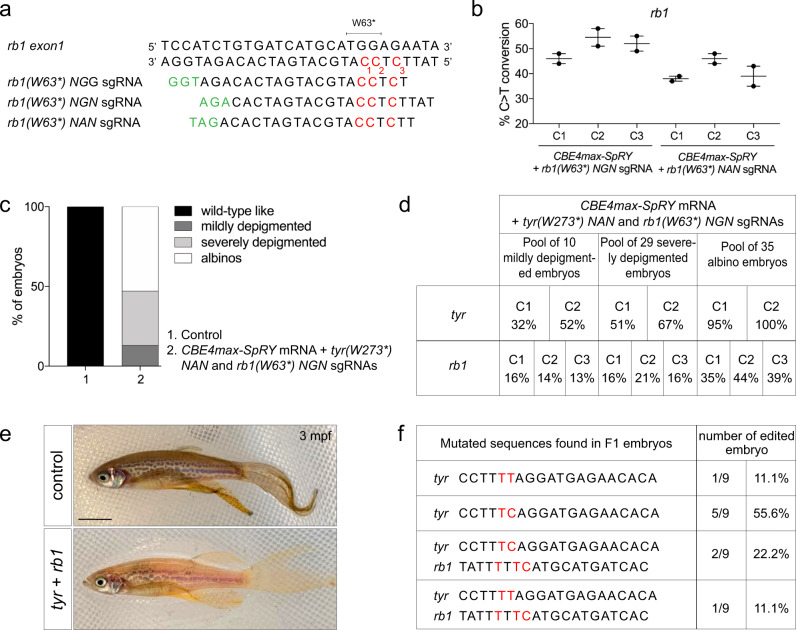
Germline transmission of two mutations generated simultaneously using NAN and NGN PAMs. **a** Targeted genomic sequence of the *rb1* tumor suppressor gene and the 3 different sgRNAs used to introduce the W63* mutation. For each sgRNA, the targeted Cs are in red and the PAM sequence is in green. **b** C-to-T conversion efficiency for each targeted Cs in 2 pools of 9 embryos injected with *CBE4max-SpRY* mRNA and rb1(W63*) NGN or rb1(W63*) NAN sgRNAs. Dot plot showing the means and standard errors of the mean (SEM). **c** Proportion of the 4 groups based on the pigmentation lack defects described in Fig. 1c for embryos injected with the *CBE4max-SpRY* mRNA, the tyr(W273*)NAN, and the rb1(W63*)NGN sgRNA (column 2, 74 embryos in total). **d** C-to-T conversion efficiency for each targeted Cs and each pool of embryos showing pigmentation defects presented in **c** for the *tyrosinase* and *rb1* genes. The efficiencies have been calculated using EditR tool^42^ and chromatograms from Sanger sequencing of PCR products. **e** Lateral view of a 3 months F0 fish injected at one-cell stage embryo with the *CBE4max-SpRY* mRNA, the tyr(W273*)NAN, and the rb1(W63*)NGN sgRNAs and showing pigmentation defects. Scale bar = 5 mm. **f** Sequenced *tyr* and *rb1* loci of 9 F1 single embryos from the founder fish in **e**. Six embryos out of 9 were single edited for *tyrosinase* and 3 embryos out of 9 were double edited for *tyrosinase* and *rb1*.

In order to explore the efficiency of the near PAM-less CBE variant in zebrafish, we decided to target the *tyrosinase* and *slc45a2* genes, respectively encoding the enzyme catalyzing the production of melanin^21^ and a solute carrier necessary for the production of melanin^22^. These two genes are thus necessary for pigment formation and mutations in the human genes are found to be linked to oculocutaneous albinism^23^. We aimed at introducing a premature STOP codon in these genes in order to have a phenotypical read out of the base conversion efficiency through the visualization of a lack of pigmentation in the larvae. We designed 3 different RNA guides upstream, respectively, of an NGG, NAN, and NGN PAM in order to introduce the W273* mutation in Tyrosinase (Fig. 1a) and 2 different RNA guides upstream of a NAN PAM in order to introduce the W121* or the R123* mutation in Slc45a2 (Fig. 1b). Upon independent injections of these sgRNAs with the *AncBE4max* mRNA for the NGG sgRNA and the C*BE4max-SpRY* mRNA for the NGG, NAN, and NGN sgRNAs, the larvae showed a range of pigmentation defects. We divided these phenotypes into four groups depending on the severity of the pigmentation defects: wild-type like, mildly depigmented, severely depigmented, and albino. Representative pictures of 2 days post-fertilization (2 dpf) larvae for each group are illustrated in Fig. 1c.

Upon the injection of the tyr(W273*) NGG sgRNA and the *AncBE4max* mRNA, we obtained 50% of depigmented larvae with a small proportion of severely affected larvae whereas surprisingly almost 100% of the injected fish with the *CBE4max-SpRY* mRNA were depigmented (Fig. 1d, columns 2 and 3). Next, using the tyr(W273*) NAN sgRNA and the CBE4max-SpRY, we obtained 100% of injected larvae showing pigmentation defects with almost 50% exhibiting a total lack of pigmentation (Fig. 1d, column 4). Finally, with the NGN PAM sgRNA, 50% of the injected larvae were poorly depigmented, the base editing efficiency reaching only 20% based on Sanger sequencing analyses (Fig. 1d columns 5, 1 f C1, Supplementary Fig. 1a). Remarkably in the pool of 9 albino larvae from the injection of the tyr(W273*) NAN sgRNA and *CBE4max-SpRY* mRNA, we obtained 100% of C-to-T conversion for the C2 (16 bp away from the PAM) and 95% for the C1 (15 bp away from the PAM) by Sanger sequencing analyses made on entire PCR (Fig. 1f and Supplementary Fig. 1a). We did not obtain this C-to-T efficiency neither using the tyr(W273*) NGN sgRNA nor the AncBE4max with the tyr(W273*) NGG sgRNA (Fig. 1d, f and Supplementary Fig. 1a). We speculate that the difference in base conversion efficiency is due to the distance of the C from the PAM or to the sgRNA sequence for which the shift of one DNA base would drastically impact the gene-editing efficiency (Fig. 1a). For the mutagenesis of *slc45a2*, we obtained the highest efficiency using the sgRNA designed for generating the W121* mutation using NAN PAM (Fig. 1e). Indeed, upon the injection of the *CBE4max-SpRY* mRNA and the slc45a2(W121*) sgRNA, 40% of the larvae were severely depigmented and 19.3% were albinos whereas only mildly depigmented larvae were observed through the injection of the slc45a2(R123*) sgRNA (Fig. 1e, f and Supplementary Fig. 1b). In order to test if we can use the CBE4max-SpRY with NYN PAMs, we designed another sgRNA to introduce the W121* mutation in Slc45a2 using NTN PAM (Supplementary Fig. 2a). Upon the injection of this sgRNA together with the *CBE4max-SpRY* mRNA, partially depigmented larvae were obtained but no albino, and the base editing efficiency reached 55% by Sanger sequencing (Supplementary Fig. 2b, c).

Moreover, 4 F0 adult fish mutated for *tyrosinase* and with pigmentation defects were screened and they all transmitted the mutated alleles to the offspring with a high transmission rate of up to 100% (Supplementary Fig. 3a, b). As for the *tyrosinase* mutants, we screened 7 F0 adult fish injected with the *CBE4max-SpRY* mRNA and the slc45a2(W121*) sgRNA. The 5 F0 fish with pigmentation defects transmitted the mutant alleles to the offspring (Supplementary Fig. 3a, c) whereas we did not detect the mutant allele in the F1 analyzed embryos from the 2 F0 fish with normal pigmentation. The screening was performed by Sanger sequencing of PCR products of the *tyrosinase* and *slc45a2* regions in random single F1 embryos from an outcross of each founder and no unwanted mutations were identified. We could also observe that the *tyrosinase* Founder 1 and the *slc45a2* Founders 1 and 2 transmitted the mutation to 100% of the offspring (Supplementary Fig. 3b, c). Moreover, by crossing 2 founders, *tyrosinase* Founder 1 and 2 (shown in supplementary Fig. 3), we were able to obtain 51.9% of albino larvae (*n* = 28/54) and by crossing the *slc45a2* Founder 1 and Founder 3, 51.1% of the larvae were albinos (*n* = 47/92) (Supplementary Fig. 3d). Together these results highlight the CBE4max-SpRY as a very powerful CBE variant, showing that for many targets this CBE could be a better choice than the classical CBEs and it could edit with a very high efficiency using NRN PAMs in zebrafish.

### Simultaneous base editing of two different genes

To test whether we can take advantage of the high flexibility and efficacy of the CBE4max-SpRY variant in zebrafish for multiplex base editing, we targeted two loci at the same time using a sgRNA upstream of an NGN PAM and one upstream of a NAN PAM.

In order to target the *retinoblastoma1* (*rb1*) gene with a higher efficiency than previously using the rb1(W63*)NGG sgRNA (Table 1), we designed two sgRNAs, one upstream of an NGN PAM (rb1(W63*)NGN sgRNA) and the second upstream of an NAN PAM (rb1(W63*)NAN sgRNA) (Fig. 2a). After injection into one-cell stage of each sgRNAs with the *CBE4max-SpRY* mRNA, we sequenced two pools of 9 injected embryos. With both sgRNAs, we obtained high base editing efficiency rates, up to 58% of efficiency with the NGN PAM and up to 48% of efficiency with the NAN PAM (Fig. 2b). Additionally, we noticed that another C was also edited, leading to the R64K mutation but 3’ to the introduced premature STOP codon (Fig. 2a, b, C3). Using the AncBE4max CBE, this C was at 19 bp from the NGG PAM and the base conversion was not detectable by Sanger sequencing (Table 1) whereas here we show that this C can be converted up to 55% of efficiency (Fig. 2c). This last result shows that for some C base targets, thanks to the PAM flexibility of the CBE4-SpRY, we can now increase the C-to-T conversion efficacy using other sgRNAs compared to the use of the classical AncBE4max (Table 1).

We next injected the *CBE4max-SpRY* mRNA and the two synthetic tyr(W273*) NAN and rb1(W63*) NGN sgRNAs together into the cell at one-cell stage. Among the injected embryos, 100% showed pigmentation defects and at least 50% exhibited a total absence of pigmentation (Fig. 2c). It can be noted that this proportion is almost the same as the one obtained after the use of the tyr(W273*) NAN sgRNA alone and the *CBE4max-SpRY* mRNA (Fig. 1d, column 4), meaning that the addition of a second guide did not affect the base conversion efficacy at the *tyrosinase* locus. Sequencing of the two loci was performed on the three different pools of larvae separated according to the severity of their pigmentation defects. As expected, the severity of the pigmentation phenotype reflected the base conversion efficiency at the *tyrosinase* locus (Fig. 2d). In particular, we found that in the pool of 35 albino larvae, we could no longer detect the wild-type C by Sanger sequencing, suggesting 100% of C-to-T conversion in the *tyrosinase* gene. Remarkably, in the albino pool, up to 44% of C-to-T conversion of the *rb1* gene was detected, revealing that double *tyrosinase* and *rb1* mutations were present in a large proportion of cells (Fig. 2d), while in mildly depigmented zebrafish, base editing of *rb1* was up to 16%. In addition, we found that both mutations were transmitted to the offspring with high transmission rates, as shown by screening the progeny of only one F0 adult fish (Fig. 2e, f). We obtained different combinations of mutations, and we showed that some F1 embryos were mutated for the *tyrosinase* gene alone. At the same time, we noticed that some embryos were mutated for different Cs of the editing windows for each gene (Fig. 2f). With these results, we could demonstrate that, using this approach, it is now possible to mutate simultaneously two different genes with high efficiency by combining two different PAM sequences in zebrafish, NAN, and NGN PAMs. Additionally, we can observe that the highest efficiency for *rb1* mutation was found in the albino larvae which also have the highest mutation rate for *tyrosinase* as measured by Sanger sequencing.

### Co-selection strategy to prescreen phenotypically the most edited larvae

**Fig. 3:**
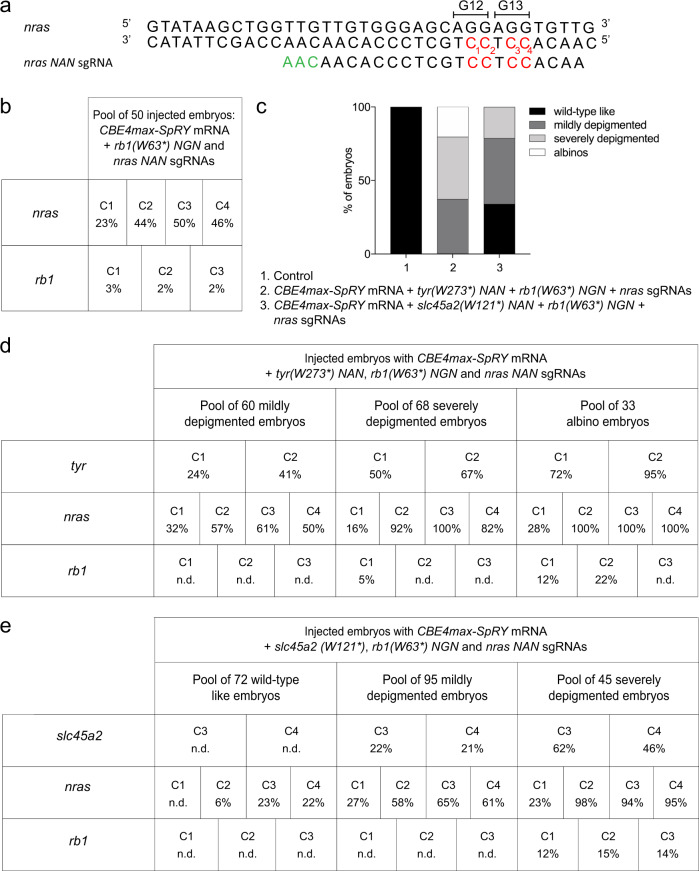
Base editing co-selection method. **a** Targeted genomic sequence of the *nras* oncogene and the sgRNA used to introduce the activating mutation. The targeted Cs are in red and the PAM sequence is in green. **b** C-to-T conversion efficiency for each targeted Cs in *nras* and *rb1* genes in a pool of 50 embryos injected with *CBE4max-SpRY* mRNA and *nras NAN* and *rb1* (W63*) NGN sgRNAs. The efficiencies have been calculated using EditR tool^42^ and chromatograms from Sanger sequencing of PCR products. **c** Proportion of the 4 groups based on the pigmentation defects described in Fig. 1c for the embryos injected with the *CBE4max-SpRY* mRNA, the tyr(W273*)NAN, nras NAA and rb1(W63*)NGN sgRNAs (column 2, 161 embryos in total) and with the *CBE4max-SpRY* mRNA, the slc45a2(W121*), nras NAA and rb1(W63*)NGN sgRNAs (column 3, 212 embryos in total). **d**, **e** C-to-T conversion efficiency for each targeted Cs and each pool of embryos showing pigmentation defects presented in Fig. 1b for the *tyrosinase*, *nras*, and *rb1* genes (**d**) and the *slc45a2*, *nras*, and *rb1* genes (**e**). The albino embryos are the most edited for the three different loci. The efficiencies have been calculated using EditR tool^42^ and chromatograms from Sanger sequencing of PCR products. N.d. = no base editing detected using EditR tool.

The efficiency of CBE4max-SpRY achieved here above opens the possibility to perform multiplex mutagenesis in zebrafish. Such experiments, however, usually involve time-consuming screening to obtain a founder carrying all the desired mutations or long crossing protocols to genetically combine multiple mutations identified in different animals. For this reason, we have decided to take advantage of the high base editing rate of the tyrW273*(NAN) and slc45a2(W121*) sgRNAs and developed a method for co-selection of base editing to rapidly identify the most edited F0 embryos following *CBE4max-SpRY* mRNA injections. We first injected the rb1(W63*)NGN and *nras NAN* sgRNAs (Fig. 3a) with the near PAM-less *CBE4max-SpRY* mRNA and found C-to-T conversion rates up to 3% at *rb1* and 50% at *nras* targets in a pool of 50 injected embryos as measured by Sanger sequencing (Fig. 3b). Moreover, at the *nras* locus we can observe that the C base which is 12 bases away from the PAM has been edited at 23% efficiency, and the C which is 18 bases away from the PAM has not been edited on the contrary to what we usually observe using the other CBE4 variants (Fig. 3b). From these observations, we can speculate that the editing windows of the CBE4max-SpRY is slightly different than the usual [−19, −13 bp] PAM window and that with the use of this CBE it is possible to target the C bases which are at - 12 bp from the PAM. We then added to the same micro-injection mix the tyr(W273*)NAN sgRNA or the slc45a2(W121*)NAN sgRNA and obtained larvae showing pigmentation defects that we split into four groups as above (Figs. 1c and 3c). For each injection, by sequencing the 3 loci in each pool of larvae, we could show that the editing efficiency is higher or lower to the same extent in the 3 targeted loci (Fig. 3d, e). For the co-selection by targeting *tyrosinase*, we could observe by Sanger sequencing analyses that the mildly depigmented larvae were edited up to 41% for *tyr*, 61% for *nras*, and no detectable mutation for *rb1*, whereas the albino larvae were edited up to 95% for *tyr*, 100% for *nras* and 22% for *rb1* (Fig. 3d). For the co-selection by targeting *slc45a2*, the mildly depigmented larvae were edited up to 22% for *slc45a2*, 65% for *nras*, and no detectable mutation for *rb1*, whereas the severely depigmented larvae were edited up to 62% for *slc45a2*, 98% for *nras*, and 15% for *rb1* (Fig. 3e). Base editing co-selection strategies were recently demonstrated in cultured cells^24^ and have not been reported in animals so far. Using the tyr(W273*) NAN or slc45a2(W121*) NAN sgRNAs, we show here a powerful strategy to readily obtain the most efficiently C base edited larvae at targeted loci of interest by simply selecting for the less pigmented larvae resulting from co-targeting the genes necessary for the pigmentation.

### Modeling genetic diseases with combinations of mutations

**Fig. 4:**
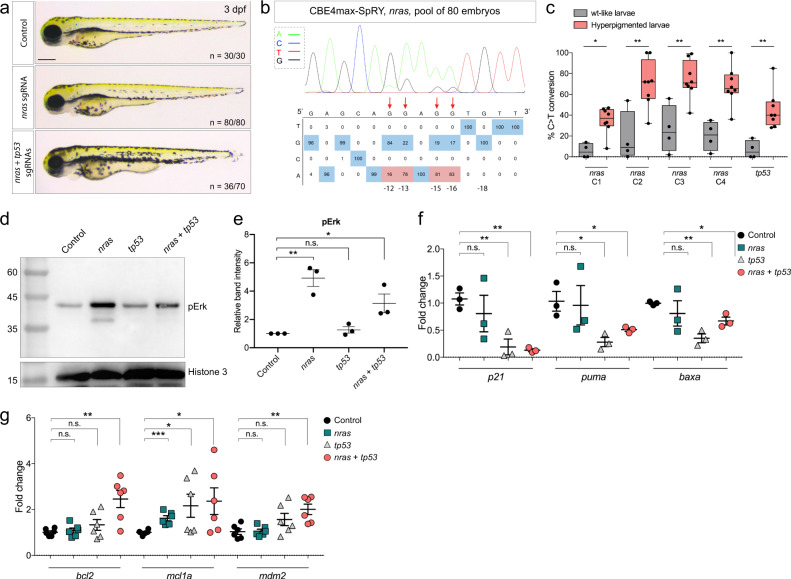
Endogenous activation of *nras* oncogene and knock-out of *tp53* tumor-suppressor gene led to an increase in melanocyte numbers. **a** Lateral view of 3 dpf larvae. The injected embryos edited only for *nras* do not present any defects, whereas 50% of the injected embryos targeted for *nras* and *tp53* were hyperpigmented. Scale bar = 100 µm. **b** DNA sequencing chromatogram of the targeted *nras* gene from a pool of 80 injected embryos with the *CBE4max-SpRY* mRNA and the *nras NAN* sgRNA. C-to-T conversion shows 83% of efficiency for the C in position 16, 81% for the C in position 15, 78% for the C in position 13, and 16% for the C in position 12 bp away from the PAM. Numbers in the boxes represent the percentage of each base at that sequence position. In red are highlighted the base substitutions introduced by base editing while the original bases are in blue. The color code of the chromatogram is indicated in the upper left corner (Adenine green, Cytosine blue, Thymine red, Guanine black). The distance from the PAM sequence of the targeted C base is indicated below the chromatogram. The efficiencies have been calculated using EditR software^42^ and chromatograms from Sanger sequencing of PCR products. **c** Box plot showing the base editing efficiency for *nras* and *tp53* genes for the 4 “wt-like” larvae and the 8 larvae with increased pigmentation from Supplementary Fig. 4. Statistical significance was determined using a two-tailed Mann–Whitney test. *p* value = 0.0162 (*nras* C1), 0.0081 (*nras* C2), 0.0081 (*nras* C3), 0.0040 (*nras* C4), 0.0020 (*tp53*). The boxes range from the 25th to 75th percentiles. The box plot whiskers down to the minimum and up to the maximum value, the center shows the median, and each individual value is represented by a point. **d** Western blot made on 3 dpf control larvae, *nras* targeted larvae, *tp53* targeted larvae or *nras* and *tp53* targeted larvae using *CBE4max-SpRY* mRNA and antibodies targeting phosphorylated ERK1/2 (pERK) and Histone 3 as loading control. The molecular weight is in kDa. Source data are provided as a Source data file. **e** Dot plot showing the means and standard errors of the mean (SEM) of the relative band intensity of 3 independent western blots as seen in **d**. Statistical significance was determined using a two-tailed unpaired *t* test. *p* value= 0.0027 (**, control vs *nras*), 0.3160 (n.s., control vs *tp53*), 0.0313 (*, control vs *tp53* + *nras*). **f**, **g** Dot plots showing the means and SEM of quantitative real-time PCR of *p21*, *puma*, *baxa* (**f**) and of *bcl2*, *mcl1a*, *mdm2* (**g**) expression levels in 3 dpf control larvae, *nras* targeted larvae, *tp53* targeted larvae or *nras* and *tp53* targeted larvae using *CBE4max-SpRY* mRNA. Statistical significance was determined using a two-tailed unpaired *t* test. For *p21*, *p* value = 0.4881 (n.s., control vs *nras*), 0.0082 (**, control *vs tp53*), 0.0011 (**, control vs *nras* + *tp53*). For *puma*, *p* value = 0.8653 (n.s., control vs *nras*), 0.0196 (*, control vs *tp53*), 0.0465 (*, control vs *nras* + *tp53*). For *baxa*, *p* value = 0.4695 (n.s., control vs *nras*), 0.0015 (**, control vs *tp53*), 0.0121 (*, control vs *nras* + *tp53*). For *bcl2*, *p* value = 0.5810 (n.s., control vs *nras*), 0.2163 (n.s., control vs *tp53*), 0.0033 (**, control vs *nras* + *tp53*). For *mcl1a*, *p* value = 0.0004 (***, control vs *nras*), 0.0445 (*, control vs *tp53*), 0.0423 (*, control vs *nras* + *tp53*). For *mdm2*, *p* value= 0.8956 (n.s., control vs *nras*), 0.1014 (n.s., control vs *tp53*), 0.0037 (**, control vs *nras* + *tp53*). *n* = 3 for (**f**) and *n* = 6 for (**g**) independent experiments.

We next investigated the ability of CBE4max-SpRY to introduce in zebrafish mutations found in human cancers. The zebrafish has become powerful in vivo model to study a high variety of human cancer types^16–19^. However, studies to assess the effects of mutations in oncogenes in the zebrafish model have relied so far on transgenic approaches that express human oncogenes using tissue-specific or constitutive promoters. In order to more accurately mimic the impact of cancer somatic mutations that cause the majority of human cancers, we decided to use the CBE4max-SpRY to generate combinations of mutations in endogenous zebrafish genes preserving their normal transcriptional regulation. To test this innovative approach, we aimed at generating an activating mutation in the *nras* gene combined with loss-of-function mutation in the *tp53* tumor-suppressor gene. We thus injected into one-cell stage embryos the *CBE4max-SpRY* mRNA, *nras NAN*, and *tp53*(Q170*) sgRNAs. We found that 50% of the double injected fish showed an over-all increase of pigmentation at 3 dpf (*n* = 36/70), a phenotype never seen upon separate injection of each sgRNA alone nor in the stable tp53 mutant embryos^25^ (Fig. 4a). To verify that the absence of phenotype when injecting only *nras NAN* sgRNA is not due to low efficiency of gene editing, we sequenced a pool of 80 injected embryos and we detected high efficiency of base conversion (Fig. 4b and Supplementary Fig. 4). We next randomly selected 4 embryos presenting a pigmentation similar to control embryos and 8 embryos showing an increase of pigmentation after the injection of the sgRNAs targeting *nras* and *tp53* genes. After sequencing both loci, we found indeed a correlation between the hyperpigmentation phenotype and the efficiency of the multi-target approach, as embryos with an increase of pigmentation had more base editing events for *nras* and *tp53* genes than normally pigmented larvae (Fig. 4c). In order to validate the effect of the activating mutation in Nras at the protein level, we checked whether the MAP kinase pathway downstream of Nras signaling is activated by a western blot analysis for phosphorylated ERK1/2^15^ (Fig. 4d). We could detect a significant increase of phosphorylated ERK when *nras* is targeted, suggesting an activation of the MAPK cascade downstream of Nras (Fig. 4d, e). We then analyzed the level of expression of several genes downstream of the transcription factor p53: *p21*, *puma*, and *baxa* (Fig. 4f). By RT-qPCR analysis, a significant decrease of expression for these 3 genes was observed in larvae when *tp53* was targeted, suggesting successful targeting of p53 (Fig. 4f). Moreover, we observed a significant increase in the expression of two anti-apoptotic genes, *bcl2* and *mcl1a*, and for *mdm2* coding for a negative regulator of p53 in larvae in which *nras* and *tp53* were targeted (Fig. 4g). These gene expression changes suggest that the simultaneous mutations in *nras* and *tp53* may prevent apoptosis, allowing cells expressing oncogenic Nras, and depleted of tp53, to survive, and perhaps proliferate, upon oncogenic transformation. Although both G12 and G13 amino acids in Nras are found mutated in human developing melanoma, the patients carry a single mutation. We thus aimed at targeting only the G12 in order to get a zebrafish model closer to human genetic events. We designed sgRNAs targeting a locus upstream of NCN PAMs (Supplementary Fig. 2d). Upon independent injections of each sgRNA with the *CBE4max-SpRY* mRNA, we could detect base editing by Sanger sequencing, reaching 82% of efficiency in a single embryo (Supplementary Fig. 2e, f). This proves that we can generate precise base editing using the CBE4max-SpRY and NCN PAM in zebrafish.

Moreover, we showed that simultaneous activation of endogenous *nras* oncogene and knock-out of *tp53* tumor suppressor gene leads to an increase of melanocyte numbers in zebrafish larvae, early evidence of abnormal melanocyte growth which could lead to melanoma formation (Fig. 4). Indeed, it has been reported that fish over-expressing human mutant HRAS oncogene in melanocytes were hyperpigmented at 3 dpf and developed melanoma at the adult stage^15^. Moreover, other reports using zebrafish transgenic lines have suggested a role of p53 and Ras oncogenes in melanoma formation^26,27^. We have developed here a hyper-pigmentation zebrafish model by generating endogenous activating mutation in *nras* oncogene and loss-of-function mutation in *tp53* tumor-suppressor gene.

### NGS analyses of base editing purity and off-targets

**Fig. 5:**
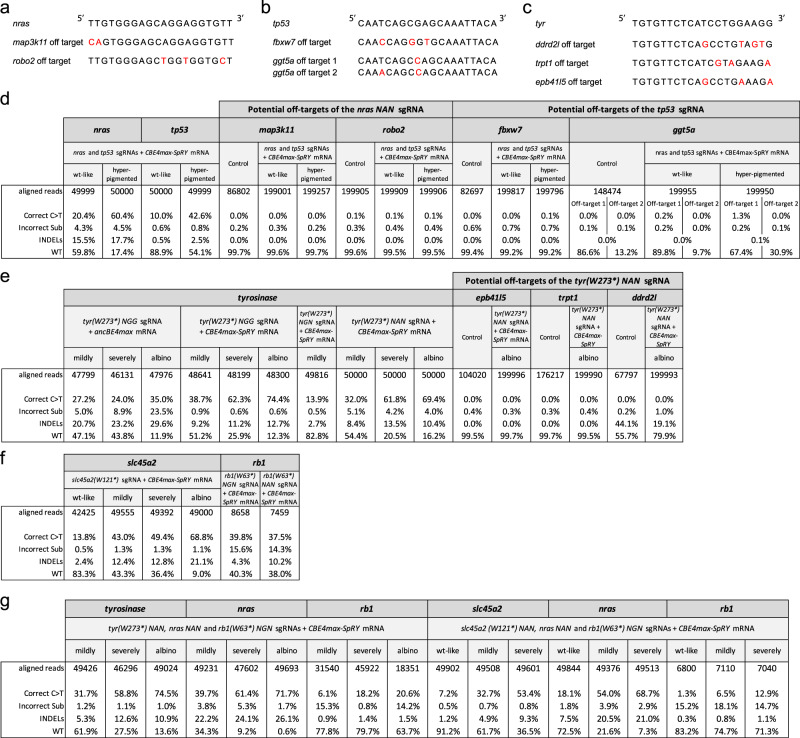
On-target and potential off-target analyses by NGS sequencing. **a**–**c** Potential off-target sequences with mismatches in red of the *nras NAN* sgRNA (**a**), *tp53* sgRNA (**b**), and tyr(W273*) NAN sgRNA (**c**). **d**–**g** NGS sequencing was made on pools of embryos with at least 6800 analyzed reads for each condition. NGS analyses of on-targets are also shown in Supplementary Fig. 5.

In order to analyze the base editing specificity of the CBE4max-SpRY variant in zebrafish, we performed NGS sequencing on 5 different loci of 38 different conditions in total (Fig. 5 and Supplementary Fig. 5). We could first show that as previously observed using Sanger sequencing, the hyper-pigmented larvae were more gene edited for *nras* and *tp53* than the wild-type like larvae (Fig. 5d and Supplementary Fig. 5). In the larvae in which *tyrosinase* or *slc45a2* gene was targeted, correlations between the base editing efficiency and the severity of the pigmentation defect were also shown, albino larvae have the highest mutation rate (Fig. 5e–g and Supplementary Fig. 5). Also, the correlations for the co-selection strategies were confirmed, the albino embryos have the highest base editing rate for all the targeted loci (Fig. 5g and Supplementary Fig. 5). However, although the NGS analyses confirmed a high rate of base editing using the near PAM-less base editor and non-NGG PAMs, it also revealed the generation of unwanted on target substitutions and/or INDELs ranging from 1.1% to 29.4% (Fig. 5 and Supplementary Fig. 5). Using the tyr(W273*) NGG sgRNA, we compared the editing products generated with AncBE4max or CBE4max-SpRY base editor and we found a higher unwanted mutation rate when using the AncBE4max. Indeed, in the albino larvae 53.1% of unwanted mutations were detected using AncBE4max vs 13.3% using CBE4max-SpRY, whereas 35 and 74,4%, respectively, of the expected C to T conversion were obtained (Fig. 5e and Supplementary Fig. 5).

In addition, we also performed NGS sequencing for two potential non-intergenic off-targets of *nras* and *tp53* sgRNAs and 3 potential non-intergenic off-targets of tyr(W273*) NAN sgRNA, identified using the CRISPOR webtool^28^ as the sequences presenting the fewest mismatches with NRN PAMs (Fig. 5a–c). For all the loci except *ggt5a*, we did not detect higher rates of mutant sequences in the injected larvae than in the control ones which were not injected (Fig. 5d, e). We found a SNP at the *ggt5a* locus which makes the *ggt5a* locus an off-target of *tp53* sgRNA with 2 mismatches instead of 1 mismatch for some alleles (Fig. 5b). Interestingly, in the hyperpigmented larvae which were base edited at 42.6% on the *tp53* gene, we could detect a base editing rate of only 1.3% on the *ggt5a* locus carrying 1 mismatch whereas we did not detect any mutation on the alleles with 2 mismatches, suggesting a high fidelity of the base editor system (Fig. 5d).

## Discussion

While the BE technology is emerging as a revolutionary method to introduce precise single mutations in the genome, the presence of the NGG PAM sequence at a suitable distance from the targeted base, defining the base editing window, is necessary and often a constraint. This has restricted its potential applications as the absence of the PAM makes the CBE unusable for many targets of interest. Here, we addressed these limitations in zebrafish by testing several base editor variants recognizing other PAM sequences. We unfortunately did not obtain any C-to-T conversions in zebrafish embryos using the previously published xCas9-BE4^20^ and only a few with the CBE4max-SpG^14^ (Table 1). Nevertheless, we could introduce point mutations with a remarkably high-efficiency rate using the CBE4max-SpRY variant, a recently described near PAM-less CBE variant engineered and validated in cultured cells but never reported working in an animal model so far^14^. We thus significantly expand the base conversion possibilities in zebrafish and are open to the possibility to convert C bases which could not be targeted so far. Our results demonstrate that the CBE4max-SpRY can be highly efficient (Figs. 2d, e, 3b, and 4c). We screened 4 F0 fish for the *tyrosinase* mutation and 5 F0 fish for the *slc45a2* mutation that transmitted the correct mutations to the offspring with a high germline transmission rate (Supplementary Fig. 3). The latter results are particularly remarkable as such a high-efficiency rate has never been reached previously in zebrafish, even with the use of the classical CBEs recognizing the NGG PAM^9–12^. For one of our targets, the CBE4max-SpRY was even more efficient than the AncBE4max (Fig. 1d and Supplementary Fig. 1a). This could be due, in part, to the fact that the CBE4max-SpRY might have a slightly different editing window that the usual PAM [-19, -13 bp] window as we show base editing for the C12 and no conversion for the C18 in *nras* targeting (Fig. 4b). Moreover, our NGS analyses revealed a higher unwanted mutation rate with the AncBE4max than with the CBE4max-SpRY for the mutagenesis of the *tyrosinase* gene (Fig. 5e). This high amount of unwanted mutations has not been reported in the previous studies using non-NGS approaches such as single clones or F1 embryos analyses where they showed up to 4% of unwanted mutations^10,11^. This observation suggests that the unwanted mutation rate found could be highly locus dependent or that its detection differs based on the sequencing approach used. By analyzing other on-target loci by NGS sequencing, we observed in a few conditions unexpected rates of unwanted mutations using the CBE4max-SpRY (Fig. 5). These unwanted mutations were not detected when analyzing the F1 embryos from our 3 different stable lines (10 founders) which carried the correct C > T conversions in *rb1*, *tyrosinase*, or *slc45a2* genes (Fig. 2e, f and Supplementary Fig. 3). Nevertheless, this rate of non-C > T mutations needs to be considered when designing experiments, and the most rigorous way for deep phenotype analyses should still rely on the generation of a stable line. The fusion of the bacteriophage gam domain to the CBE4max-SpRY could also reduce INDEL formation as has been the case with the classical BE4^7^. Nevertheless, for more than ¾ of the 38 conditions tested, the expected C > T conversion was the most frequent mutation and we thus propose that the transient mutagenesis by base editor can still be used as a first candidate screen. For instance, in the analysis of *nras* and *tp53* targeted larvae, we obtained similar unwanted mutation rates between the wt-like and hyperpigmented larvae (nras: 19.8%, tp53: 1.1 vs nras: 22.2%, tp53: 3.6%) and a high difference for the correct C > T rate (nras: 20.4%, tp53: 10.0 vs nras: 60.4%, tp53: 42.6%). We could also functionally validate the knock-out of Tp53 and the specific activation of Nras that cannot be due to unwanted mutations (Fig. 4). The latter results and the PAM flexibility of the CBE4max-SpRY now allow to test several sgRNAs for the mutation of interest and play with the C base localization within the editing window to increase the C-to-T conversion efficacy compared to the use of the classical AncBE4max. These properties allow also to exclude other Cs present in the editing window to avoid the generation of other unwanted mutations near the targeted C. We furthermore demonstrated that using NAN and NGN PAMs we were able to precisely and simultaneously perform the Tyr (W273*) and Rb1 (W63*) mutations with high-efficiency rates and both mutations were transmitted to the germline (Fig. 2). In this line, we also achieved simultaneously 3 different and precise mutagenesis events using 3 different PAM sequences (Fig. 3). This ability is very useful in zebrafish if several mutations need to be introduced in order to model a human genetic disease such as cancer, especially if some mutations are located on the same chromosome. Different CRISPR co-selection methods have been engineered in *Drosophila*, *C. elegans*, and cultured cells in order to phenotypically detect and enrich the cells or animals in which more mutagenesis events are taking place, by adding an sgRNA conferring a phenotypical read-out if the mutagenesis occurred^29–33^. Base editing co-selection strategies were recently demonstrated in cultured cells^24^ but have not been reported in animals so far. Moreover, these time-saving strategies have never been developed in zebrafish. Here we describe a co-selection method for base editing in zebrafish to phenotypically prescreen injected embryos based on the detection of pigmentation defects. The method is based on the addition of the tyr(W273*) NAN sgRNA or the slc45a2(W121*) sgRNA to the micro-injection mix. Importantly mutations in the *tyr* gene seem to be semi-viable, probably due to defects in the function of the visual system^34^, whereas the loss-of-function mutation in the *slc45a2* gene does not affect viability and fertility in zebrafish and represents therefore a better co-selection gene^21^. We indeed have shown in this study that using this strategy we could select the most depigmented embryos which were the most C-to-T converted for *nras* and *rb1* genes (Fig. 3b, c). We also developed a zebrafish model combining activating mutations of *nras* oncogene and knock-out of *tp53* tumor suppressor gene causing an increase of melanocytes (Fig. 4), a clear melanoma predisposing phenotype. Indeed, fish over-expressing human mutated HRAS oncogene in melanocytes were hyperpigmented at 3dpf and developed melanoma at the adult stage^15^. We further validated the effects of these mutations at the protein level by western blot and RT-qPCR analyses. The RT-qPCR analyses also showed that pro-apoptotic genes (*baxa* and *puma*) are downregulated and anti-apoptotic genes (*bcl2* and *mcl1a*) are upregulated, suggesting that this combination of mutations may prevent apoptosis of melanocytes expressing edited oncogenic nras (Fig. 4g, f) and allow cancer cell survival and proliferation. Moreover, the upregulation of the *mdm2* gene coding for a negative regulator of p53 could favor the repression of residual p53 activity in the double mutant in addition to the activation of Nras which confer a growth advantage through pERK signaling pathway (Fig. 4). Compared to the other existing genome editing technologies, the cytosine base editor approach seems to be the most efficient to perform C:G to T:A conversion, and the design of the sgRNA is more straightforward than for the pegRNAs and the knock-in DNA templates for alternative approaches^35–38^. Moreover, although the presence of INDELs has been revealed by the NGS sequencing for several loci, this frequency remains much lower than the one of the expected mutations in contrast to what was reported for prime editing and HDR-based knock-in strategies^35–38^. However, the base editor is limited to the type of modifications and the presence of bystander Cs can be problematic as they can be converted as well. This last problem is nevertheless mitigated by our results with CBE4max-SpRY thanks to the high flexibility of sgRNA design that in most cases can be shifted to target only the wanted mutation. Based on our results, we conclude that CBE technology is a more efficient, easier to design, and time-saving approach to introduce a specific C:G to T:A mutation into the genome and should be considered as an alternative for prime editing and HDR-based knock-in techniques. Moreover, while our manuscript was under review, the Cas9-SpRY has been shown to be efficient for classical knock-out approaches generating INDELS in zebrafish^39^. The use of the CBE4max-SpRY is expected to increase the number of potential off-targets compared to the classical base editors as it is highly flexible on the type of PAM used; however, the NGS analyses revealed a good fidelity of the CBE4max-SpRY.Indeed, the presence of only one mismatch on the off-target site decreases drastically the efficiency of base editing and for the other analyzed off-targets no mutations were detected (Fig. 5d). It is nevertheless important to verify potential off-targets for each targeted locus. In addition, the CBE4max-SpRY allows also the design of different sgRNAs to target the same mutation and the selection of the one which has the least amount of predicted off-targets. The high efficiencies of CBE4max-SpRY obtained in this study and the possibility to precisely mutate simultaneously several genes using different PAMs pave the way for future applications in a tissue-specific manner and for genetic disease modeling. For example, it could be implemented in the MAZERATI (Modeling Approach in Zebrafish for Rapid Tumor Initiation) system^40^ in order to rapidly model and study in vivo combinations of endogenous mutations occurring in complex multigenic disorders. Finally, the high flexibility and efficiency of our method to induce a combination of specific mutations will allow to rapidly create zebrafish cancer models combining the precise set of mutations found in individual patients. In the long term, these models could be used for rapid and patient-specific modeling to be used in drug screening for advanced personalized medicine^17,41^.

## Methods

### Fish lines and husbandry

Zebrafish (*Danio rerio*) were maintained at 28 °C on a 14 h light/10 h dark cycle. Fish were housed in the animal facility of our laboratory which was built according to the respective local animal welfare standards. All animal procedures were performed in accordance with French and European Union animal welfare guidelines with protocols approved by the committee on ethics of animal experimentation of Sorbonne Université (APAFIS#21323-2019062416186982).

### Molecular cloning

To generate the *pCS2*+*_CBE4max-SpG* and the *pCS2*+*_CBE4max-SpRY* plasmids, each *CBE4max-SpG* and *CBE4max-SpRY* sequence has been inserted into *pCS2*+ plasmid linearized with EcoRI using the Gibson Assembly Cloning Kit (New England Biolabs). The fragment has been amplified using the primers F-5’-TGCAGGATCCCATCGATTCGGCCACCATGAAACGGACAG-3’ and R-5’-TAGAGGCTCGAGAGGCCTTGTCAGACTTTCCTCTTCTTCTTGG-3’) from the *pCAG-CBE4max-SpG-P2A-EGFP* plasmid (Addgene plasmid #139998)^14^ and from the *pCAG-CBE4max-SpRY-P2A-EGFP* plasmid (Addgene plasmid #139999)^14^.

### mRNA synthesis

*pCS2*+*_CBE4max-SpG* plasmid has been used to generate *CBE4max-SpG* mRNA in vitro. *pCS2*+*_CBE4max-SpRY* plasmid has been used to generate *CBE4max-SpRY* mRNA in vitro. Each plasmid was linearized with NotI restriction enzyme and mRNAs were synthesized by in vitro transcription with 1 µL of GTP from the kit added to the mix and lithium chloride precipitation (using the mMESSAGE mMACHINE sp6 Ultra kit #AM1340, Ambion).

*pCMV_ancBE4max*^8^ (a gift from David Liu _ Addgene plasmid #112094) has been linearized using AvrII restriction enzyme; mRNAs were synthesized by in vitro transcription with 1 µL of GTP from the kit added to the mix and lithium chloride precipitation (using the mMESSAGE mMACHINE T7 Ultra kit #AM1345, Ambion).

### sgRNA design

The sequenceParser.py python script^12^ was used to design *tyr* and *slc45a2* sgRNAs. All the synthetic sgRNAs were synthesized by IDT as Alt-R® CRISPR-Cas9 crRNA. Prior injections, 2 µL of the crRNA (100 pmol/µL) and 2 µL of Alt-R® CRISPR-Cas9 tracrRNA (100 pmol/µL) from IDT were incubated at 95 °C for 5 min, cooled down at room temperature, and then kept in ice. The crRNA used is presented in Supplementary Fig. 6.

### Micro-injection

To make the mutagenesis with base editing, a mix of 1 nL of *CBE* mRNA and synthetic sgRNAs was injected into the cell at one-cell stage zebrafish embryos. For the single mutagenesis, the final concentration was 600 ng/μL for *CBE* mRNA and 43 pmol/μL for sgRNA. For the double *rb1* and *tyr* mutations, the final concentration was 600 ng/μL for *CBE* mRNA and 21 pmol/μL for each sgRNAs. For the double *rb1* and *nras* mutations, the final concentration was 600 ng/μL for *CBE* mRNA and 8,6 pmol/μL for *nras* sgRNA and 34.4 pmol/μL for *rb1* sgRNA. For the double *p53* and *nras* mutations, the final concentration was 600 ng/μL for *CBE* mRNA and 8,6 pmol/μL for *nras* sgRNA and 34.4 pmol/μL for t*p53* sgRNA. For the *tyr or slc45a2*, *nras* and *rb1* mutations, the final concentration was 600 ng/μL for *CBE* mRNA, 8.6 pmol/μL for *nras* sgRNA and 8.6 pmol/μL for *tyr* or *slc45a2* sgRNA and 25.8 pmol/μL for *rb1* sgRNA.

### Whole-embryo DNA sequencing

For genomic DNA extraction, embryos were digested for 1 h at 55 °C in 0.5 mL lysis buffer (10 mM Tris, pH 8.0, 10 mM NaCl, 10 mM EDTA, and 2% SDS) with proteinase K (0.17 mg/mL, Roche Diagnostics) and inactivated 10 min at 95 °C. To sequence and check for frequency of mutations, each target genomic locus was PCR-amplified using Phusion High-Fidelity DNA polymerase (Thermo Scientific). The primers used are presented in Supplementary Fig. 7.

The amplified DNAs have been extracted on an agarose gel and purified (using the PCR clean-up gel extraction kit #740609.50, Macherey-Nagel) and the Sanger sequencings have been performed by Eurofins. The sequences were analyzed using ApE software and quantifications of the mutation rates done using EditR 1.0.10 online tool (https://moriaritylab.shinyapps.io/editr_v10/)^42^.

### qPCR

For gene expression analysis, total RNA was extracted from twenty 5dpf larvae in triplicate (for each experimental group: non-injected, *nras* sgRNA, *tp53* sgRNA, or NRAS + p53 sgRNAs with *CBE4max-SpRY* mRNA) with TRIzol reagent (Invitrogen). Total RNA was cleaned up using RNeasy Mini Kit (Qiagen) following the manufacturer’s instructions and treated twice with DNase I (1 unit/μg RNA, Qiagen). The RNA concentration was quantified using nanodrop2000 (Thermo Fisher) and VILO superscript KIT (Thermo Fisher) was used for First-strand cDNA synthesis according to the manufacturer’s protocol. qRT-PCR was performed using SMOBio qPCR Syber Green Mix (TQI-201- PCR Biosystem) using a standard amplification protocol. The primers used are presented in Supplementary Fig. 8.

Data analysis was performed with Microsoft Excel and GraphPad/Prism 7. In all cases, each qPCR was performed with triplicate samples and repeated with at least three independent samples. Data are expressed as fold changes compared to controls.

### Western blot analysis

Western blot analysis was carried out using standard methods. Briefly, 5 dpf larvae (n = 20, x 3 biological replicates) of each experimental group (non-injected, *nras* sgRNA, *tp53* sgRNA or NRAS + p53 sgRNAs with *CBE4max-SpRY* mRNA) were lysed on ice with lysis buffer (150 mmol/L NaCl, 50 mmol/L TRIS pH 7.4, 0.25% NP40 (Sigma-Aldrich; 11754599001), 1 mM EDTA, 0.1% Triton X100, 0.1% SDS, 0.1 mg/mL phenylmethylsulfonyl fluoride, protease cocktail inhibitor (Sigma-Aldrich; 04693159001), 50 mmol/L NaF, and 10 mmol/L Na3O4V. For Western blots, equal protein concentrations were resolved via 12% SDS–PAGE and transferred to Biorad PVDF membranes. Antibodies used: anti-pERK (Cell Signalling Technology, cat. no. 9101S, diluted 1:1000), anti-H3 (Abcam, cat. no. 1791, 1:1000) Goat anti-Rabbit IgG (HRP, diluted 1:1000) Abcam, cat. no. 6721 ECL Western Blotting Substrate (GeneTex, Trident fento Western HRP substrate, cat. no. GTX14698) was added before detection with BioRad Chemidoc XRF+. Band intensity of pERK signals was normalized to levels of H3 signal, using the Biorad Image Lab software.

### NGS sequencing of genomic DNA samples

Genomic sites of interest were amplified from genomic DNA samples. Briefly, primers were designed to generate between 228 and 313 bp amplicons using Phusion High-Fidelity DNA polymerase (Thermo Scientific). The primers used are presented in Supplementary Fig. 9.

The amplified DNAs have been extracted on an agarose gel and purified (using the PCR clean-up gel extraction kit #740609.50, Macherey-Nagel).

Raw data of the NGS sequencing organization is listed in Supplementary Fig. 10.

Illumina adapters and barcodes were added to amplicon pools by ligation and sequenced on an Illumina NextSeq (NovaSeq PE250, Novogene company). Alignment of amplicon sequences to a reference sequence was performed using a custom python pipeline that was used to count nucleotide substitutions in the base editor window (both expected C:G to T:A conversions and other substitutions) and indels overlapping the spacer sequence.

### Imaging

Embryos were oriented in egg solution with an anesthetic (Tricaine 0.013%). Leica MZ10F was used to image them. Adult fish were imaged using a net and an Iphone xs. Images were analyzed and adjusted for contrast using ImageJ/FIJI version 1.0.

### Statistics and reproducibility

No statistical method was used to predetermine sample size. No data were excluded from the analyses and samples were randomized when the injection did not affect the pigmentation. For tyrosinase and slc45a2 knock-out and hyperpigmentation experiments, embryos were split into groups based on their pigmentation. A non-parametric two-sided *t* test with the Mann–Whitney correction was applied to determine significance in base editing and to analyze the relative band intensity of the western blot. A parametric two-sided *t* test was used to determine the significance of the qPCR experiments. The software used was GraphPad/Prism 7.

### Reporting summary

Further information on research design is available in the Nature Research Reporting Summary linked to this article.

## Supplementary information

Supplementary Information

Reporting Summary

## Data Availability

The NGS data have been deposited to the NCBI Sequence Read Archive database under accession PRJNA825759. The other data that support the findings of this study are available from the corresponding authors upon request. Source data are provided with this paper.

## Source data

Source Data

## Code availability

The code for the custom python pipeline for NGS data analysis is available upon request.
