# Supplementary material for: Disease modeling by efficient genome editing using a near PAM-less base editor in vivo
Source: Nat Commun. 2022 Jun 15;13:3435. doi: 10.1038/s41467-022-31172-z (PMC9198099; doi:10.1038/s41467-022-31172-z)
Supplement: Supplementary file 1 — Supplementary Information [file 41467_2022_31172_MOESM1_ESM.pdf]

Supplementary informations

Disease modeling by efficient genome editing using a near PAM-less base editor *in vivo*.

(Rosello *et al.)*

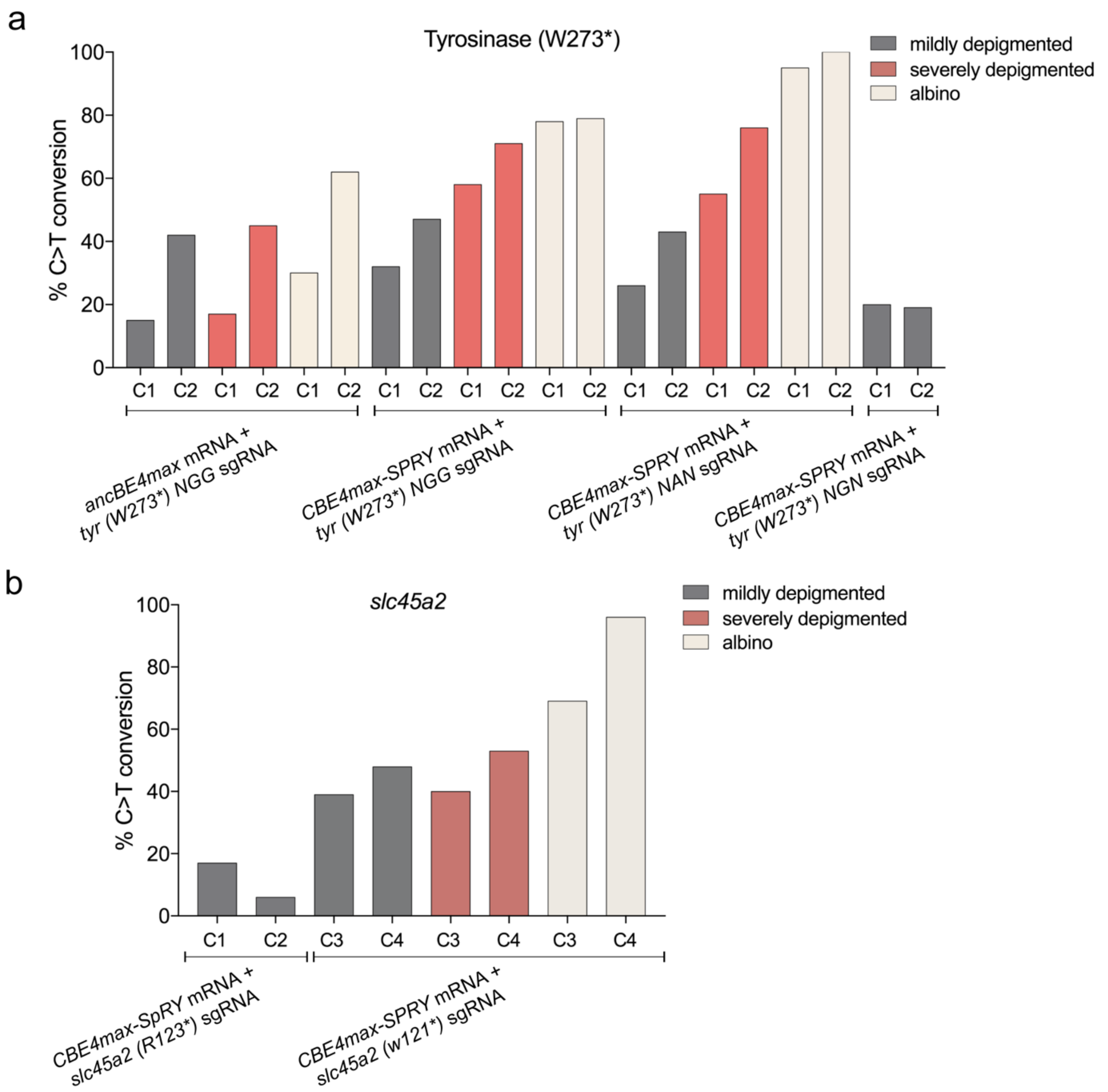

Supplementary Figure 1. Efficient base conversions of *tyrosinase* and *slc45a2* genes with CBE4max-SpRY and a NAN PAM.
a-b. Histograms representing the C-to-T conversion efficiency (found in Fig. 1f) of each targeted Cs and each pool of embryos showing pigmentation defects presented in Fig. 1d and Fig. 1e by targeting *tyrosinase* (a) or *slc45a2* (b) genes. The efficiencies have been calculated using EditR tool[41] and chromatograms from Sanger sequencing made on entire PCR products.
a. column 1 and 2: 7 embryos, column 3 and 4: 3 embryos, column 5 and 6: 1 embryo, column 7 and 8: 35 embryos, column 9 and 10: 31 embryos, column 11 and 12: 7

embryos, column 13 and 14: 5 embryos, column 15 and 16: 14 embryos, column 17 and 18: 9 embryos, column 19 and 20: 5 embryos.
b. column 1 and 2: 100 embryos, column 3 and 4: 37 embryos, column 5 and 6: 60 embryo, column 7 and 8: 29 embryos.

a

W121*

slc45a2 5' CTACTGTAGGTCGTCATGGGGCCGAAGGAGACCGTACAT 3'
3' GATGACATCCAGCAGTACCCCGGCTTCCTCTGGCATGTA 5'
(3 4 5)

slc45a2(W121)* sgRNA 1 GATGACATCCAGCAGTACCCCGG

slc45a2(W121)* sgRNA 2 ATGACATCCAGCAGTACCCCGGC

b

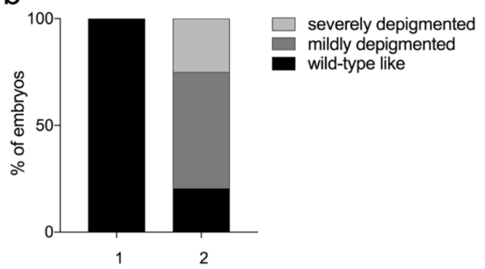

1. Control
2. *CBE4max-SpRY* mRNA + *slc45a2(W121*)* sgRNA 2

c

	Injected embryos with *CBE4max-SpRY* mRNA + *slc45a2 (W121*)* sgRNA 2								
	Pool of 22 wild-type like embryos			Pool of 59 mildly depigmented embryos			Pool of 27 severely depigmented embryos		
slc45a2	C3 n.d.	C4 n.d.	C5 n.d.	C3 30%	C4 32%	C5 21%	C3 53%	C4 55%	C5 33%

d

G12 G13

nras 5' GTATAAGCTGGTTGTTGTGGGAGCAGGAGGTGTTG 3'
3' CATATTCGACCAACAACACCCTCGTCCTCCACAAC 5'
(1 2 3 4)

nras sgRNA 1 (NAN) AACAACACCCTCGTCCTCCACAA

nras sgRNA 2 (NCN) CCAACAACACCCTCGTCCTCCAC

nras sgRNA 3 (NCN) ACCAACAACACCCTCGTCCTCCA

e

	Pool of 80 embryos sgRNA 1 (NAN)				Pool of 31 embryos sgRNA 2 (NCN)				Pool of 60 embryos sgRNA 3 (NCN)			
nras CBE4max-SpRY	C1 16%	C2 78%	C3 81%	C4 83%	C1 8%	C2 12%	C3 n.d.	C4 n.d.	C1 16%	C2 17%	C3 n.d.	C4 n.d.

f

		emb. 1		emb. 2		emb. 3		emb. 4		emb. 5		emb. 6		emb. 7		emb. 8	
CBE4max-SpRY	sgRNA 2	C1 36%	C2 19%	C1 35%	C2 22%	C1 n.d.	C2 n.d.	C1 n.d.	C2 n.d.	C1 n.d.	C2 n.d.	C1 n.d.	C2 n.d.	C1 n.d.	C2 n.d.	C1 n.d.	C2 n.d.
	sgRNA 3	C1 37%	C2 36%	C1 43%	C2 42%	C1 44%	C2 41%	C1 65%	C2 65%	C1 54%	C2 50%	C1 82%	C2 80%	-	-	-	-

Supplementary Figure 2. CBE4max-SpRY edits NYN PAMs in zebrafish.
a. Targeted genomic sequence of the *slc45a2* oncogene and the sgRNAs used to introduce the W121*mutation. The targeted Cs are in red and the PAM sequence is in green. **b**. Proportion of the 4 groups based on the pigmentation lack defects described in Fig. 1c for embryos injected with the *CBE4max-SpRY* mRNA and *slc45a2(W121*)* sgRNA2 (column 2, 108 embryos in total). **c**. C-to-T conversion efficiency for each targeted Cs and each pool of embryos showing pigmentation defects presented in Supplementary Fig. 2b. The efficiencies have been calculated using EditR software[38] and chromatograms from Sanger sequencing of PCR products. n.d.= non-detectable edits by Sanger sequencing. **d**. Targeted genomic sequence of the *nras* oncogene and

the sgRNAs used to introduce the activating mutation. The targeted Cs are in red and the PAM sequence is in green. **e-f**. C-to-T conversion efficiency for each targeted Cs and pool of injected embryos (**e**) or in single embryo randomly selected (**f**) with each sgRNA targeting *nras* and *CBE4max-SpRY* mRNA. The efficiencies have been calculated using EditR software[38] and chromatograms from Sanger sequencing of PCR products. N.d.=no base editing detected using EditR tool.

a

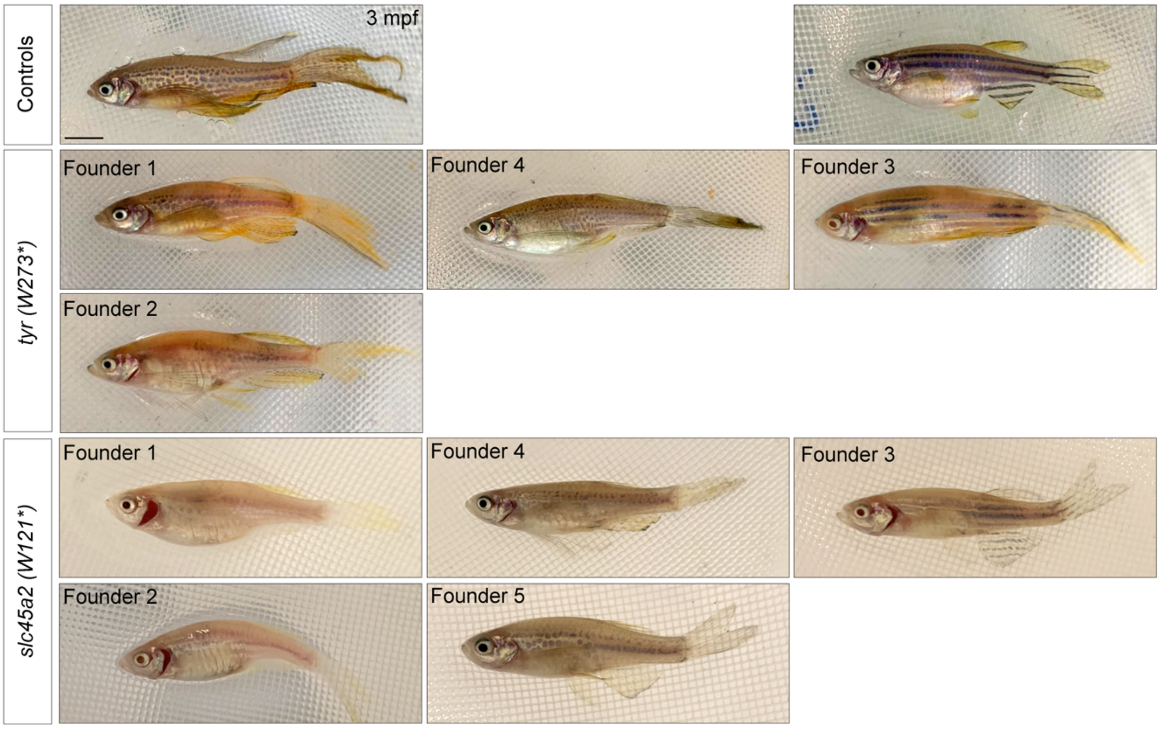

b

tyrosinase	Founder 1		Founder 2		Founder 3		Founder 4	
CCTTCCAGGATGAGAACACA	0/16	0%	3/16	18.7%	12/15	80%	2/16	87.5%
CCTTTTAGGATGAGAACACA	16/16	100%	11/16	68,8%	1/15	6.7%	2/16	12.5%
CCTTTCAGGATGAGAACACA	0/16	0%	2/16	12.5%	2/15	13.3%	0/16	0%

c

slc45a2	Founder 1		Founder 2		Founder 3		Founder 4		Founder 5	
GGCCCCATGACGACCTACAG	0/16	0%	0/8	0%	11/16	68.7%	12/15	80%	12/13	92.3%
GGCCCTATGACGACCTACAG	3/16	18.8%	2/8	25%	0/16	0%	2/15	13.3%	1/13	7.7%
GGCCTTATGACGACCTACAG	13/16	81.2%	6/8	75%	3/16	18.8%	1/15	6.7%	0/13	0%
GGCTTTATGACGACCTACAG	0/16	0%	0/16	0%	2/16	12.5%	0/15	0%	0/13	0%

d

F1 larvae from the cross of *slc45a2(W121*)* Founder 1 and *slc45a2(W121*)* Founder 3

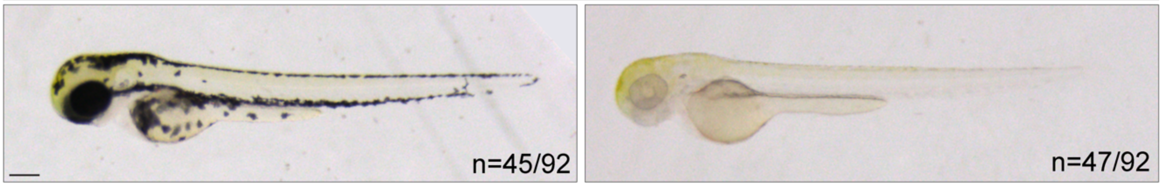

Supplementary Figure 3. High germline transmission rate.
a. Lateral view of a 3 months F0 fish injected at one-cell stage with the *CBE4max-SpRY* mRNA and the *tyr(W273*)NAN* or *slc45a2(W121*)* sgRNA and showing pigmentation lack defect. Scale bar= 5 mm. **b.** Sequenced *tyrosinase* locus of F1 single embryos randomly chosen from an outcross of each founder. 16 embryos out of 16 were edited for *tyrosinase* for the Founder 1, 13 embryos out of 16 for the Founder 2, 3 embryos out of 15 for the Founder 3 and 2 embryos out of 16 for the Founder 4. **c.** Sequenced *slc45a2* locus of F1 single embryos randomly chosen from an outcross of each founder. 16 embryos out of 16 were edited for *slc45a2* for the Founder 1 and 2, 5 embryos out of 16 for the Founder 3, 3 embryos out of 15 for the Founder 4 and 1 embryo out of 13 for the Founder 5. **d**. Lateral view of 3 dpf F1 larvae from the cross of the *slc45a2* Founder 1 and Founder 3. Scale bar= 100µm.

Larvae injected with *CBE4max-SpRY* mRNA + *nras* and *tp53* sgRNAs	*nras*				*tp53*
	C1	C2	C3	C4	
"wt-like" larvae 1	n.d.	6%	29%	27%	9%
"wt-like" larvae 2	n.d.	n.d.	2%	3%	n.d.
"wt-like" larvae 3	9%	12%	18%	15%	n.d.
"wt-like" larvae 4	14%	54%	56%	35%	18%
larvae 1 with increased pigmentation	29%	55%	68%	63%	28%
larvae 2 with increased pigmentation	41%	59%	66%	61%	40%
larvae 3 with increased pigmentation	8%	32%	42%	36%	29%
larvae 4 with increased pigmentation	47%	100%	97%	80%	49%
larvae 5 with increased pigmentation	33%	72%	70%	67%	36%
larvae 6 with increased pigmentation	32%	73%	80%	75%	54%
larvae 7 with increased pigmentation	46%	72%	72%	64%	40%
larvae 8 with increased pigmentation	44%	100%	99%	100%	85%

Supplementary Fig. 4. Hyperpigmented embryos present a higher base editing efficiency for *nras* and *tp53* than the wild-type like embryos.
C-to-T conversion efficiency for each targeted Cs in *nras* and *tp53* genes in single embryos injected with *CBE4max-SpRY* mRNA and *nras NAN* and *tp53* (*Q170*)* sgRNAs. 4 single larvae which did not show an increase of pigmentation named as "wt-like" larvae and 8 single larvae showing an increase of pigmentation have been sequenced. N.d.=no base editing detected using EditR tool.

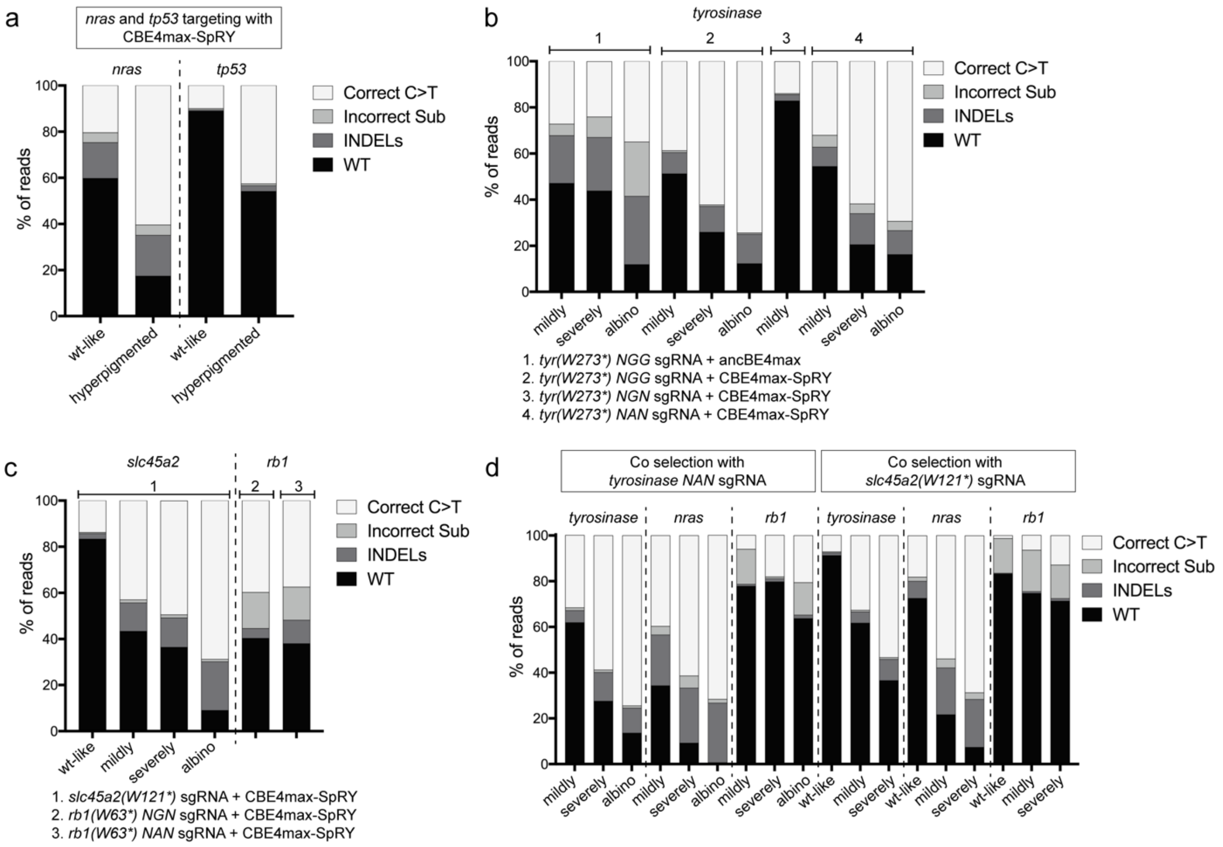

Supplementary Figure 5. NGS sequencing analysis of some on-targets.

a-d. Proportion of correct C>T conversion, incorrect substitution, INDELs and wild-type sequences reads founds after NGS sequencing analyses of *nras*, *tp53*, *tyrosinase*, *slc45a2* and *rb1* genes (quantification found in Fig. 5).

a. Embryos injected with *nras* and *tp53* sgRNAs together with the *CBE4max-SpRY* mRNA.

b. Embryos injected with each *tyrosinase(W273*)* sgRNAs together with the *AncBE4max* (1) or *CBE4max-SpRY* mRNAs (2, 3 and 4).

c. Embryos injected with *CBE4max-SpRY* mRNA and *slc45a2(W121*)* sgRNA (1), *rb1(W63*) NGN* sgRNA (2) or *rb1(W63*) NAN* sgRNA (3).

d. Embryos injected with *CBE4max-SpRY* mRNA and *tyrosinase(W273*) NAN, nras* and *rb1(W63*) NAN* sgRNAs (co selection with *tyrosinase NAN* sgRNA). Embryos injected with *CBE4max-SpRY* mRNA and *slc45a2(W121*), nras* and *rb1(W63*) NAN* sgRNAs (co selection with *slc45a2(W121*)* sgRNA).

	crRNA sequences (5'-3')
bap1	GACTCAGCAAGAATCAGGCC
cbl	AGTTCCAGTCTGGCATGTTG
tek	GGGTCAGGAGTTCCCCCATA
dmd	ACACTCCAGCTGAACAGCTC
rb1(W63) NGG*	TCTCCATGCATGATCACAGA
rb1(W63) NGN*	TATTCTCCATGCATGATCAC
rb1(W63) NAN*	ATTCTCCATGCATGATCACA
tp53	CAATCAGCGAGCAAATTACA
nras NAN	AACACCTCCTGCTCCCACAA
nras NCN 1	CACCTCCTGCTCCCACAACA
nras NCN 2	ACCTCCTGCTCCCACAACAA
tyr(W273) NGG*	CTTCCAGGATGAGAACACAG
tyr (W273) NGN*	TTCCAGGATGAGAACACAGA
tyr (W273) NAN*	CCTTCCAGGATGAGAACACA
slc45a2 (W121)*	GGCCCCATGACGACCTACAG
slc45a2 (R123)*	TGGGGCCGAAGGAGACCGTA
slc45a2 (W121) NTN*	CGGCCCCATGACGACCTACA

Supplementary Figure 6. crRNA sequences used in the study.

	Forward Primer (5'-3')	Reverse Primer (5'-3')
tyr	ATCGGGTGTATCTGCTGTTTTGG	CCATACCGCCCCTAGAACTAACATT
rb1	TCTGTCAACTGTTGTTTTTCCAGAC	CAATAAAAAGACAAGCTCCCCACTG
nras	CCTTTTCTCTCTTTTTGTCTGGGTG	CGCAATCTCACGTTAATTGTAGTGT
tp53	ATATCTTGTCTGTTTTCTCCCTGCT	GTCCTACAAAAAGGCTGTGACATAC
bap1	TTGTTTATTTTTCAGGACCATGGGG	CACCTGAAGGTATTGGTGTTTCTTG
tek	ATCTCAGACGTGACTCTGGTGAAC	TTCCTGTAGCATCTTGTAGGTGTAG
cbl	TTCCCATCACAAAGACAAGCCATTA	CTGTTCTGGAATCGAGAGTAGAGG
dmd	AGGGCTCCTTCCTTTTTCTGTTTAT	TGATCGAGTTTTGATGATTTCTCCG
slc45a2	AACCATGACTCTTCTTACTGAGGAC	GACCCTGAAACTCATCTACTTCCTT

Supplementary Figure 7. Sequences of the primers used to amplify the targeted loci for Sanger sequencing.

	Forward Primer (5'-3')	Reverse Primer (5'-3')
puma	GAACACACGGGTTACAAAAGAC	GAAAATTCCCAGAGTCTGTAAGT G
baxa	GTGGCGCTTTTCTACTTTGC	GGAAAACTCCGACTGTCTGC
p21	ACCACTAGAGGGCGGAGACT	CTGGGGTTTTCTCCACTTCA
mdm2	AGCTGATTGGCTTCCAGAAA	GGTAGTGGCAGGAGATGGAA
mcl1a	AAAACAAGAGCTGGCATGGG	CCAAAAGACCATGACGGACAAC
bcl2	AACGCCTGTGAAGATGAGGA	TGCAGTCTGGGTCATGTGAT
rsp11 (housekeeping)	ACAGAAATGCCCCTTCACTG	GCCTCTTCTCAAAACGGTTG

Supplementary Figure 8. Sequences of the primers used for the qPCR analysis.

	Forward Primer (5'-3')	Reverse Primer (5'-3')
nras	TAAACTGTGAGTCAAACTGTGTGTG	CGCAATCTCACGTTAATTGTAGTGT
tp53	ATATCTTGTCTGTTTTCTCCCTGCT	GTCCTACAAAAAGGCTGTGACATAC
map3k11	TGCCGTGAGGAGGAGCTGAA	CATGCTGATCTTCTCTCCGTTCTTC
robo2	CCCTACACCTGATGCCACTTTTT	GTATATAGCAGGAGGTGACAGTGAG
fbxw7	CAGTACTGTGACGGTTGGGTTTAG	CACAATGCCATGAACTGTTATAGGG
ggt5a	GTAAATTTGTGACCAAGGTTTTGGC	GAGTATGATGTGAGGACAAACTGGA
tyr	ATCGGGTGTATCTGCTGTTTTGG	CCATACCGCCCCTAGAACTAACATT
epb41l5	CAGCATGTTCAAGATTGACTCGCAAA	TTGTGTGAACAAATGCGCTATGAG
trpt1	TTCTGATTTGTTGGTCTTTCAGAGC	ACAAGAATTGCAGACAACAGAGATG
ddrd2l	ACACTTCTCAGTGTTTTGGTTGACA	GCTGATGATGTCTGTTTCTGCAT
slc45a2	AAACTGGTGAGCTACAAGTAGAGAG	GCCAAGCTAAATGAGCGTTATTAGT
rb1	TCTGTCAACTGTTGTTTTTCCAGAC	CAATAAAAAGACAAGCTCCCCACTG

Supplementary Figure 9. Sequences of the primers used to amplify the targeted loci for NGS sequencing.

Conditions	fastq files	Target gene
control	ddrd_tyr_1_200k.fq	*ddrd2l*
tyrosinase targeted with NAN PAM and CBE4max-SpRY, albino	ddrd_tyr_4_200k.fq	*ddrd2l*
control	epb_tyr_1_200k.fq	*epb41l5*
tyrosinase targeted with NAN PAM and CBE4max-SpRY, albino	epb_tyr_4_200k.fq	*epb41l5*
control	trpt_tyr_1_200k.fq	*trpt1*
tyrosinase targeted with NAN PAM and CBE4max-SpRY, albino	trpt_tyr_4_200k.fq	*trpt1*
control	fbxw_p53_1_200k.fastq	*fbxw7*
nras and p53 targeted, wt-like	fbxw_p53_2_200k.fastq	*fbxw7*
nras and p53 targeted, hyperpigmented	fbxw_p53_3_200k.fastq	*fbxw7*
control	ggt_p53_1_200k.fq	*ggt5a*
nras and p53 targeted, wt-like	ggt_p53_2_200k.fq	*ggt5a*
nras and p53 targeted, hyperpigmented	ggt_p53_3_200k.fq	*ggt5a*
control	map_nras_1_200k.fq	*map3k11*
nras and p53 targeted, wt-like	map_nras_2_200k.fq	*map3k11*
nras and p53 targeted, hyperpigmented	map_nras_3_200k.fq	*map3k11*
control	robo_nras_1_200k.fq	*robo2*
nras and p53 targeted, wt-like	robo_nras_2_200k.fq	*robo2*
nras and p53 targeted, hyperpigmented	robo_nras_3_200k.fq	*robo2*
coselection with slc45a2, wt-like	nras_7_50k.fastq	*nras*
coselection with slc45a2, mildly depigmented	nras_8_50k.fastq	*nras*
coselection with slc45a2, severely depigmented	nras_9_50k.fastq	*nras*
nras and p53 targeted, wt-like	nras_10_50k.fastq	*nras*
nras and p53 targeted, hyperpigmented	nras_11_50k.fastq	*nras*
coselection with tyrosinase, mildly depigmented	nras_15_50k.fastq	*nras*
coselection with tyrosinase, severely depigmented	nras_16_50k.fastq	*nras*
coselection with tyrosinase, albinos	nras_17_50k.fastq	*nras*
rb1 with NGN PAM and CBE4max-SpRY	rb1_5_50k.fastq	*rb1*
rb1 with NAN PAM and CBE4max-SpRY	rb1_6_50k.fastq	*rb1*
coselection with slc45a2, wt-like	rb1_7_50k.fastq	*rb1*
coselection with slc45a2, mildly depigmented	rb1_8_50k.fastq	*rb1*
coselection with slc45a2, severely depigmented	rb1_9_50k.fastq	*rb1*
coselection with tyrosinase, mildly depigmented	rb1_15_50k.fastq	*rb1*
coselection with tyrosinase, severely depigmented	rb1_16_50k.fastq	*rb1*
coselection with tyrosinase, albinos	rb1_17_50k.fastq	*rb1*
nras and p53 targeted, wt-like	p53_10_50k.fastq	*tp53*
nras and p53 targeted, hyperpigmented	p53_11_50k.fastq	*tp53*
coselection with slc45a2, wt-like	slc45a2_7_50k.fastq	*slc45a2*
coselection with slc45a2, mildly depigmented	slc45a2_8_50k.fastq	*slc45a2*
coselection with slc45a2, severely depigmented	slc45a2_9_50k.fastq	*slc45a2*
slc45a2 targeted alone, wt-like	slc45a2_10_50k.fastq	*slc45a2*

slc45a2 targeted alone, group mildly depigmented	slc45a2_11_50k.fastq	*slc45a2*
slc45a2 targeted alone, severely depigmented	slc45a2_12_50k.fastq	*slc45a2*
slc45a2 targeted alone, albino	slc45a2_13_50k.fastq	*slc45a2*
tyrosinase targeted with NGG PAM and AncBE4max, mildly depigmented	tyr_5_50k.fastq	*tyrosinase*
tyrosinase targeted with NGG PAM and AncBE4max, severely depigmented	tyr_6_50k.fastq	*tyrosinase*
tyrosinase targeted with NGG PAM and AncBE4max, albino	tyr_7_50k.fastq	*tyrosinase*
tyrosinase targeted with NGG PAM and CBE4max-SpRY, mildly depigmented	tyr_8_50k.fastq	*tyrosinase*
tyrosinase targeted with NGG PAM and CBE4max-SpRY, severely depigmented	tyr_9_50k.fastq	*tyrosinase*
tyrosinase targeted with NGG PAM and CBE4max-SpRY, albino	tyr_10_50k.fastq	*tyrosinase*
tyrosinase targeted with NGN PAM and CBE4max-SpRY, mildly depigmented	tyr_11_50k.fastq	*tyrosinase*
tyrosinase targeted with NAN PAM and CBE4max-SpRY, mildly depigmented	tyr_12_50k.fastq	*tyrosinase*
tyrosinase targeted with NAN PAM and CBE4max-SpRY, severely depigmented	tyr_13_50k.fastq	*tyrosinase*
tyrosinase targeted with NAN PAM and CBE4max-SpRY, albino	tyr_14_50k.fastq	*tyrosinase*
coselection with tyrosinase, mildly depigmented	tyr_15_50k.fastq	*tyrosinase*
coselection with tyrosinase, severely depigmented	tyr_16_50k.fastq	*tyrosinase*
coselection with tyrosinase, albinos	tyr_17_50k.fastq	*tyrosinase*

Supplementary Figure 10. Raw data of the NGS sequencing.
The fastq files are available on NCBI Sequence Read Archive database under accession PRJNA825759.
