# Supplementary material for: Disease modeling by efficient genome editing using a near PAM-less base editor in vivo
Source: Nat Commun. 2022 Jun 15;13:3435. doi: 10.1038/s41467-022-31172-z (PMC9198099; doi:10.1038/s41467-022-31172-z)
Supplement: Supplementary file 2 — Reporting Summary [file 41467_2022_31172_MOESM2_ESM.pdf]

nature portfolio

Corresponding author(s): Filippo Del Bene
Jean-Paul Concordet

Last updated by author(s): May 27, 2022

Reporting Summary

Nature Portfolio wishes to improve the reproducibility of the work that we publish. This form provides structure for consistency and transparency in reporting. For further information on Nature Portfolio policies, see our Editorial Policies and the Editorial Policy Checklist.

Statistics

For all statistical analyses, confirm that the following items are present in the figure legend, table legend, main text, or Methods section.

n/a	Confirmed	
☐	☒	The exact sample size (*n*) for each experimental group/condition, given as a discrete number and unit of measurement
☐	☒	A statement on whether measurements were taken from distinct samples or whether the same sample was measured repeatedly
☐	☒	The statistical test(s) used AND whether they are one- or two-sided<br>*Only common tests should be described solely by name; describe more complex techniques in the Methods section.*
☒	☐	A description of all covariates tested
☒	☐	A description of any assumptions or corrections, such as tests of normality and adjustment for multiple comparisons
☐	☒	A full description of the statistical parameters including central tendency (e.g. means) or other basic estimates (e.g. regression coefficient) AND variation (e.g. standard deviation) or associated estimates of uncertainty (e.g. confidence intervals)
☐	☒	For null hypothesis testing, the test statistic (e.g. *F*, *t*, *r*) with confidence intervals, effect sizes, degrees of freedom and *P* value noted<br>*Give P values as exact values whenever suitable.*
☒	☐	For Bayesian analysis, information on the choice of priors and Markov chain Monte Carlo settings
☒	☐	For hierarchical and complex designs, identification of the appropriate level for tests and full reporting of outcomes
☒	☐	Estimates of effect sizes (e.g. Cohen's *d*, Pearson's *r*), indicating how they were calculated

Our web collection on statistics for biologists contains articles on many of the points above.

Software and code

Policy information about availability of computer code

Data collection	no software was used
Data analysis	ImageJ/FIJI version 1.0<br>EditR 1.0.10 (https://moriaritylab.shinyapps.io/editr_v10/)<br>GraphPad/Prism 7<br>ApE v2.0.53c

For manuscripts utilizing custom algorithms or software that are central to the research but not yet described in published literature, software must be made available to editors and reviewers. We strongly encourage code deposition in a community repository (e.g. GitHub). See the Nature Portfolio guidelines for submitting code & software for further information.

Data

Policy information about availability of data

All manuscripts must include a data availability statement. This statement should provide the following information, where applicable:

- Accession codes, unique identifiers, or web links for publicly available datasets
- A description of any restrictions on data availability
- For clinical datasets or third party data, please ensure that the statement adheres to our policy

We deposited the NGS sequencing data on NCBI (BioProject ID: PRJNA825759) and created a Source Data file.

Field-specific reporting

Please select the one below that is the best fit for your research. If you are not sure, read the appropriate sections before making your selection.

[x] Life sciences [ ] Behavioural & social sciences [ ] Ecological, evolutionary & environmental sciences

For a reference copy of the document with all sections, see nature.com/documents/nr-reporting-summary-flat.pdf

Life sciences study design

All studies must disclose on these points even when the disclosure is negative.

Sample size	For zebrafish experiments, sample sizes were not predetermined based on statistical methods, but were chosen according to common standards in the field. qPCR and western blots experiments where designed to reach an n larger that 3 (each samples with 20 larvae pooled).
Data exclusions	No data were excluded from the analyses
Replication	Each experiment was repeated at least twice and all attempts were successful.
Randomization	Samples were randomized when the injection did not affect the pigmentation. For tyrosinase knock-out and hyperpigmentation experiments, embryos were split into groups based on their pigmentation.
Blinding	sgRNAs were injected in a blind manner. The sequenced embryos were picked randomly except for the experiments for which groups based on pigmentation defects were done.

Reporting for specific materials, systems and methods

We require information from authors about some types of materials, experimental systems and methods used in many studies. Here, indicate whether each material, system or method listed is relevant to your study. If you are not sure if a list item applies to your research, read the appropriate section before selecting a response.

Materials & experimental systems

n/a	Involved in the study	
[ ]	[x]	Antibodies
[x]	[ ]	Eukaryotic cell lines
[x]	[ ]	Palaeontology and archaeology
[ ]	[x]	Animals and other organisms
[x]	[ ]	Human research participants
[x]	[ ]	Clinical data
[x]	[ ]	Dual use research of concern

Methods

n/a	Involved in the study	
[x]	[ ]	ChIP-seq
[x]	[ ]	Flow cytometry
[x]	[ ]	MRI-based neuroimaging

Antibodies

Antibodies used	anti-pERK (Cell Signalling Technology, cat. no. 9101S)<br>anti-H3 (Abcam, cat. no. 1791)<br>Goat anti-Rabbit IgG (HRP) Abcam, cat. no. 6721.
Validation	anti-pERK validated in zebrafish for WB according to vendor website https://www.cellsignal.com/products/primary-antibodies/phospho-p44-42-mapk-erk1-2-thr202-tyr204-antibody/9101?_=1653655595666&Ntt=9101S&tahead=true<br><br>anti-H3 validated in zebrafish for WB according to vendor website https://www.abcam.com/histone-h3-antibody-nuclear-marker-and-chip-grade-ab1791.html?productWallTab=ShowAll

Animals and other organisms

Policy information about studies involving animals; ARRIVE guidelines recommended for reporting animal research

Laboratory animals	Adult zebrafish in TL or AB background of age between 3 and 18 months where used to produce embryos. In each experiment larvae and embryos between 1 and 3 days post fertilization where used as described. Larval zebrafish were studied before the onset of sexual differentiation.
Wild animals	This study did not involve wild animals.
Field-collected samples	This study did not involve samples collected from the field.

Ethics oversight	All procedures were performed on zebrafish embryos in accordance with the European Communities Council Directive (2010/63/EU) and French law (87/848) and approved by the Sorbonne Universite´ ethic committee (Charles Darwin) and the French Ministry for research (APAFIS agreement #21323 2019062416186982).

Note that full information on the approval of the study protocol must also be provided in the manuscript.
